# Intravenous lanadelumab for the treatment of moderately ill COVID‐19 patients

**DOI:** 10.1002/bcp.70438

**Published:** 2026-01-09

**Authors:** Job J. Engel, Christine van Linge, W. Joost Wiersinga, Ilse J. E. Kouijzer, Quirijn de Mast, Robert‐Jan Hassing, Danique J. H. Huijbens, Helen L. Leavis, Roger Schutgens, Coen Maas, Kit C. B. Roes, Frank L. van de Veerdonk, Roger Brüggemann

**Affiliations:** ^1^ Department of Internal Medicine and Radboud Centre for Infectious Diseases Radboudumc Nijmegen the Netherlands; ^2^ Centre for Experimental and Molecular Medicine (CEMM) and Division of Infectious Diseases, Amsterdam University Medical Centres Amsterdam University Medical Centres‐ Location AMC, University of Amsterdam Amsterdam the Netherlands; ^3^ Department of Internal Medicine Rijnstate Hospital Arnhem the Netherlands; ^4^ Department of Rheumatology and Clinical Immunology University Medical Centre Utrecht Utrecht the Netherlands; ^5^ Centre for Benign Haematology, Thrombosis and Haemostasis, Van Creveldkliniek University Medical Centre Utrecht Utrecht the Netherlands; ^6^ Central Diagnostic Laboratory University Medical Centre Utrecht Utrecht the Netherlands; ^7^ Department of Health Evidence, Section Biostatistics Radboudumc, Radboud University Nijmegen the Netherlands; ^8^ Department of Pharmacy and Radboudumc Institute for Health Sciences Radboudumc Nijmegen the Netherlands

**Keywords:** COVID‐19, kallikrein‐kinin system, lanadelumab, plasma kallikrein, SARS‐CoV‐2

## Abstract

**Aims:**

Kallikrein‐kinin system (KKS) dysregulation is hypothesized to play a pathogenetic role in COVID‐19‐associated pulmonary oedema. To investigate the efficacy and safety of intravenous lanadelumab, a monoclonal antibody that inhibits plasma kallikrein, in COVID‐19, we conducted a phase 2, open‐label, randomized‐controlled, proof‐of‐concept, interventional trial.

**Methods:**

We recruited 40 patients hospitalized with moderate COVID‐19 and randomized them 1:1 to receive either standard‐of‐care (SoC) treatment or SoC plus intravenous lanadelumab (300 mg on days one and four). The primary outcome consisted of repeated measurements of supplemental oxygen (litres/minute) necessary to maintain a peripheral oxygen saturation (SpO_2_) ≥ 93%. Secondary outcomes included modified WHO‐CPS scores, need for high‐flow oxygen therapy or mechanical ventilation, admission to the intensive care unit, length of hospital stay and all‐cause mortality over a 14‐day period.

**Results:**

Sufficient endpoint data for the population were available for the first five days, but not for the previsioned 14‐day endpoint. Consequently, analysis of the primary endpoint was based on the first five days of treatment. Within this timeframe, lanadelumab did not significantly affect supplemental oxygen volumes. Neither treatment nor interaction between treatment and time was a significant predictor of oxygen volumes in a linear mixed model (p = 0.49 and p = 0.15, respectively). None of the secondary outcomes was significantly affected by lanadelumab. Intravenous lanadelumab was well tolerated.

**Conclusions:**

This exploratory study was evaluated using a shortened primary endpoint period. Lanadelumab showed no indication of benefit on oxygen needs or other clinical outcomes in patients with COVID‐19. Lanadelumab was well tolerated throughout the trial.

**What is already known about this subject**

- Kallikrein‐kinin system activation has been demonstrated in COVID‐19.
- Multiple kallikrein‐kinin system inhibitors have been investigated for the treatment of COVID‐19‐associated pulmonary oedema.

**What this study adds**

- This is the first clinical trial in which lanadelumab was used intravenously in patients with an active infection.
- Lanadelumab had no significant effect on supplemental oxygen requirement or other clinical outcomes in patients with COVID‐19.

## 1 INTRODUCTION

The development of pulmonary oedema in patients with COVID‐19 can lead to the requirement of supplemental oxygen and—in severe cases—acute respiratory distress syndrome.^1^ It has been hypothesized that this is due in part to local vascular leakage resulting from dysregulation of the kallikrein‐kinin system (KKS), which may be exacerbated by manipulation of angiotensin‐converting enzyme 2 (ACE2) by SARS‐CoV‐2.^2^, ^3^, ^4^, ^5^

The KKS is a proteolytic pathway whose end products are oligopeptides called kinins—such as bradykinin (BK) and Lys‐bradykinin (LBK)—which are produced through enzymatic hydrolysis of kininogen precursors. Kinins can induce inflammation, vasodilation and increased vascular permeability by binding the bradykinin B1 (B1R) and B2 receptor (B2R).^6^, ^7^, ^8^, ^9^ KKS activation in COVID‐19 is demonstrated by accumulation of bradykinin metabolites in the bronchoalveolar lavage (BAL) fluid and systemic circulation of patients with severe COVID‐19.^10^, ^11^ This has been linked to thromboinflammation and worse clinical outcomes.^12^ ACE2, which functions as the cellular receptor for SARS‐CoV‐2, likely plays a central role in COVID‐19‐associated KKS dysregulation, as it metabolizes kinin oligopeptides like des‐Arg^9^‐bradykinin (DABK) and Lys‐des‐Arg^9^‐bradykinin (LDABK).^13^, ^14^, ^15^ KKS activation and limited availability of ACE2 in the pulmonary environment could therefore contribute to pulmonary oedema in COVID‐19, and may be medically targetable during SARS‐CoV‐2 infection.^2^, ^14^, ^16^, ^17^

Multiple treatment options targeting the KKS are available. Upstream targets such as plasma kallikrein (PKa), the primary enzyme responsible for BK formation in circulating plasma, can be inhibited with the monoclonal antibody lanadelumab. Icatibant acts downstream of the KKS pathway by antagonizing the B2R.^6^, ^17^, ^18^, ^19^, ^20^, ^21^ We demonstrated in a case–control study that icatibant rapidly reduced supplemental oxygen need in moderately ill COVID‐19 patients.^22^ Similarly, three‐day icatibant treatment in addition to SoC increased clinical efficacy and reduced 28‐day mortality in a trial of 67 COVID‐19 patients.^23^ However, icatibant is short‐acting, requiring multiple injections due to its short elimination half‐life of 1.48 ± 0.35 h, and a resurgence in supplemental oxygen need after treatment was observed in several patients in our previous study.^22^, ^24^, ^25^, ^26^ Moreover, icatibant treatment had a 90% probability of offering <50% benefit on time to recovery in COVID‐19 patients admitted to the ICU in the I‐SPY COVID‐19 trial, leading to early termination of its icatibant arm.^27^ We therefore designed the present study to investigate the efficacy of blocking kinin generation using lanadelumab.

Lanadelumab is a fully human monoclonal antibody, whose main clinical use lies in the prophylaxis of angioedema attacks characteristic of hereditary angioedema (HAE). In these patients, subcutaneous lanadelumab is well tolerated and has a low potential for interactions with comedication.^19^, ^20^, ^21^ It inhibits BK generation by disrupting the hydrolysis of high‐molecular‐weight kininogen (HK) by active PKa. Lanadelumab is absorbed slowly after subcutaneous administration and has a much longer half‐life than icatibant (approximately 14.8 days).^19^ In addition, icatibant only targets the B2R, while lanadelumab affects all kinins downstream of PKa. Thus, lanadelumab should dampen the effects of HK‐derived BK on both B1R and B2R, which could be clinically relevant considering the fact that B1R expression is upregulated by pro‐inflammatory proteins such as tumour necrosis factor (TNF‐α) and interleukin‐1β (IL‐1β).^6^, ^7^, ^28^ These factors could make lanadelumab a preferable treatment for COVID‐19‐associated pulmonary oedema over icatibant, while offering a more patient‐friendly treatment regimen by circumventing the necessity for repeat treatments. In addition, while there are extensive data supporting the use of lanadelumab in HAE, there are no published studies investigating its use in pulmonary oedema specifically. This study presents the first clinical data on the efficacy of lanadelumab in that setting.

Here we describe a randomized, standard‐of‐care (SoC)‐controlled, proof‐of‐concept study including a total of 40 patients admitted to the hospital with moderate COVID‐19, of whom 20 received SoC treatment and 20 received intravenous lanadelumab in addition to SoC.

## 2 MATERIALS AND METHODS

### 2.1 Trial summary

The Covid‐Lanadelumab trial is a prospective, proof‐of‐concept, phase 2, open‐label, randomized‐controlled interventional study comparing intravenous lanadelumab in addition to SoC treatment to SoC alone for COVID‐19‐associated pulmonary oedema. The trial was conducted in four Dutch medical centres (Radboudumc, Nijmegen; Rijnstate Hospital, Arnhem; Amsterdam University Medical Centre, location Academic Medical Centre, Amsterdam; University Medical Centre Utrecht, Utrecht).

The protocol for this study was approved by the regional medical ethics committee (Medisch‐Ethische Toetsingscommissie; METC) of Radboudumc. The study was conducted in accordance with principles of Good Clinical Practice and the Declaration of Helsinki. Written informed consent was obtained from all participants. Trial execution and data entry were monitored by an independent research associate, assigned through Radboudumc pharmacy's Clinical Trials Unit. This study is registered at ClinicalTrials.gov under trial number NCT04422509.

### 2.2 Patients

Patients were eligible for inclusion according to the following criteria: (1) A positive RT‐PCR result for SARS‐CoV‐2 RNA; (2) SpO_2_ < 90% on pulse oximetry and/or a need for ≥ 3 L/min of supplemental oxygen; (3) Age 16 or above. Only patients admitted to (non‐ICU) general care wards treated with low‐flow supplemental oxygen therapy were screened for inclusion in the trial to ensure similar disease severity between randomization groups. Exclusion criteria were: (1) previous participation in this study; (2) acute myocardial or cerebral ischemic events at the time of enrolment; (3) plasma alanine aminotransferase or aspartate aminotransferase concentrations > 5x the upper limit of normal at baseline; (4) known hypersensitivity to fully human monoclonal antibodies; (5) pregnancy or breastfeeding at the time of enrolment. Additionally, if patients were using ACE‐inhibitors, angiotensin receptor blockers (ARB's), or comparable drugs intervening with the renin‐angiotensin‐aldosterone system (RAAS), they were excluded from receiving those medications during the trial.^5^, ^9^

This trial was designed to include 40 COVID‐19 patients admitted to general care wards across the abovementioned medical centres. As this was a proof‐of‐concept trial, its aim was to detect signals of potential efficacy of the study drug, allowing for future investigations. No formal sample size calculation was performed, similar to previously conducted exploratory interventional trials conducted in COVID‐19 patients.^29^, ^30^, ^31^, ^32^ A sample size of 20 patients per randomization group was considered adequate to evaluate the relevant study endpoints in an exploratory context. Furthermore, this number of inclusions was deemed feasible by the participating medical centres, given the logistical constraints of conducting this trial during the COVID‐19 pandemic.

### 2.3 Randomization and trial interventions

After inclusion, patients were electronically randomized using Castor Electronic Data Capture in a 1:1 ratio to either a control group receiving only SoC treatment or an intervention group receiving SoC plus two intravenous doses of lanadelumab. At the time this trial was conducted, SoC consisted of supplemental oxygen therapy, dexamethasone (daily dose of 6 mg for a maximum of 10 days), interleukin‐6 receptor antagonists if dexamethasone alone proved insufficient (defined as worsening clinical status, ≥6 L/min supplemental oxygen, or C‐reactive protein [CRP] > 75 mmol/L, with persisting pulmonary inflammation as the most likely cause), and prophylactically dosed anticoagulant therapy (low‐molecular‐weight heparins in most cases) in accordance with Dutch COVID‐19 treatment guidelines. ^28^, ^33^, ^34^, ^35^ Randomization was not stratified according to trial centre.

Upon inclusion, patients underwent physical examination and blood pressure, respiratory rate, SpO_2_, type of oxygen delivery system, and supplemental oxygen volume were documented. Follow‐up measurements of these respiratory parameters were performed every 24 h during the trial, or until patients were discharged from the hospital. A protocol aimed at active reduction of supplemental oxygen was used for all patients. This protocol started upon enrolment in the trial and was enacted throughout its 14‐day follow‐up period. Patients randomized to the intervention group received 300 mg of lanadelumab intravenously on days one and four of the trial. The selected intravenous dose given to study participants is the same as the dose used subcutaneously in the prophylactic setting in HAE, with the sole difference being the route of administration. Peak concentrations of intravenous lanadelumab were assumed to be reached at the end of infusion. Clearance was assumed to be identical in COVID‐19 patients as in HAE patients. Modelling and simulations performed prior to the start of this trial provided the necessary justification for the dose selection (proprietary pharmacometric data provided by the manufacturer). For safety considerations, a ramp‐up scheme was used in which the infusion rate was steadily increased over time until the full dose of lanadelumab was administered. Further details on blood sampling, the active supplemental oxygen reduction protocol, and the ramp‐up scheme can be found in the trial protocol and the supplementary methods section (Supplementary Files 1 & 2, respectively).

### 2.4 Outcome measures

The primary outcome consisted of repeated (daily) measurements of supplemental oxygen volumes necessary to maintain SpO_2_ ≥ 93% during a 14‐day follow‐up period. At all timepoints, only volumes in the low‐flow range were considered suitable for evaluation (i.e., only when a nasal cannula, nasal bridle, Venturi mask, or non‐rebreathing mask was used to deliver oxygen, up to 15 L/min). At any point when supplemental oxygen therapy was escalated to a high‐volume oxygen delivery system (e.g., high‐flow nasal cannula, non‐invasive ventilation, or mechanical ventilation), this was considered treatment failure and subsequent primary outcome data were no longer evaluated. Primary outcome data were expressed as litres of supplemental oxygen per minute. The oxygen delivery system being used was documented using an ordinal scale (1 = nasal cannula; 2 = nasal bridle; 3 = Venturi mask; 4 = non‐rebreathing mask).

The primary endpoint did not take into account the need for high‐flow oxygen therapy or mechanical ventilation. Therefore, we reported COVID‐19 severity in both treatment groups at all timepoints as a secondary outcome measure. For this, we adapted the WHO Clinical Progression Scale (WHO‐CPS), an 11‐point ordinal scale with scores ranging from 0 to 10.^36^ The WHO‐CPS requires repeated PCR testing for the presence of viral and the documentation of ventilator settings and the use of vasopressors, dialysis, or ECMO to differentiate between scores 0–2 and 7–9, respectively. Because these data were not collected as part of our trial, we chose to modify the WHO‐CPS in the following way: scores ranging from 0 to 2 were compiled into a single score (0–2), and scores ranging from 7 to 9 were likewise documented as a compiled score (7–9). An overview of this modified scale can be seen in Figure 2A. Other secondary endpoints were the need for high‐flow oxygen therapy or mechanical ventilation, the need for ICU admission, total length of hospital stay and all‐cause mortality during follow‐up. Secondary outcomes were still considered evaluable if patients required high‐flow oxygen therapy or mechanical ventilation during the trial.

Lanadelumab concentrations were determined using a validated Enzyme‐Linked Immunosorbant Assay (ELISA; according to EMA and FDA guidance documents) for quantitation in human SCAT‐169 tubes. The assay ranged from 3.1 to 400 ng/mL. Further details on pharmacokinetic sampling and analyses can be found in Supplementary File 2.

### 2.5 Statistical analysis

Descriptive and inferential statistics were performed in SPSS Statistics (IBM software). Data were visualized using Prism (GraphPad software) and Adobe Illustrator (Adobe Systems incorporated). The pharmacokinetic dataset was constructed and analysed using Phoenix WinNonlin (Phoenix software).

To evaluate the primary outcome, we performed a linear mixed model analysis of repeated supplemental oxygen volumes, where treatment group, time and an interaction term of treatment group and time were entered as fixed effects. A first‐order autoregressive covariance structure with homogenous variances (AR(1)) was used. For the secondary endpoints, different tests were used according to the dependent variables concerned. Modified WHO‐CPS scores were represented as percentages within each treatment group and visualized at key timepoints (screening and days 1, 2, 4, 7 and 14). Comparisons between randomization groups in the need for ICU admission, high‐flow oxygen therapy and mechanical ventilation during follow‐up were performed using Fisher's exact test. Total length of stay and length of stay in the ICU were compared using Mann–Whitney U tests. For all analyses, a p‐value of < 0.05 was considered statistically significant. Given the exploratory nature of this study, no correction for multiplicity was applied to statistical testing.

### 2.6 Safety outcomes

Adverse events (AEs), serious adverse events (SAEs) and suspected unexpected serious adverse reactions (SUSARs) were reported and documented in accordance with Good Clinical Practice guidelines.

### 2.7 Nomenclature of targets and ligands

Key protein targets and ligands in this article are hyperlinked to corresponding entries in https://www.guidetopharmacology.org, and are permanently archived in the Concise Guide to PHARMACOLOGY 2023/24.^5^, ^8^, ^9^, ^28^

## 3 RESULTS

### 3.1 Trial population

Between the 30th of October 2020, and the 15th of February 2021, a total of 40 patients were randomized across the participating medical centres. Table 1 shows the baseline characteristics of patients in both randomization groups. Mean age was higher in the control group than in the intervention group (63.9 years *vs*. 55.5 years, respectively). The ratio of male to female participants also differed between groups (20% female in the control group *vs*. 55% in the intervention group). Other demographic characteristics and factors relating to disease severity did not differ significantly, although median Charlson Comorbidity Index (CCI) was higher in the control group than the intervention group (3.0 *vs*. 2.0, respectively). None of the study participants had been vaccinated against SARS‐CoV‐2 at inclusion.

**TABLE 1:** Demographic and clinical patient characteristics.

Demographic characteristics	Control group (N = 20)	Intervention group (N = 20)
Age‐mean ± SD	63.9 ± 9.5	55.5 ± 13.1
65 yr. or older – no./total no. (%)	11/20 (55.0%)	7/20 (35.0%)
Male – no./total no. (%)	16/20 (80.0%)	9/20 (45.0%)
Body mass index (kg/m^2^) – mean ± SD	30.0 ± 4.3	29.8 ± 8.1
Charlson comorbidity index (CCI) – median (range)	3 (0–6)	2 (0–5)
**Clinical characteristics**		
Days from symptom onset to randomization – mean ± SD	10.6 ± 3.9	10.5 ± 3.4
SpO_2_ with supplemental oxygen upon randomization (%) – mean ± SD	93.9 ± 1.7	94.3 ± 2.4
Supplemental oxygen volume upon randomization (L/min) – mean ± SD	6.5 ± 3.0	7.3 ± 3.4
Respiratory rate upon randomization (breaths/minute) – mean ± SD	22.6 ± 6.8	21.8 ± 4.9
Type of supplemental oxygen upon randomization^a^		
Nasal cannula – no./total no. (%)	12/19 (63.2%)	10/19 (52.6%)
Nasal bridle – no./total no. (%)	1/19 (5.3%)	0/19 (0.0%)
Venturi mask – no./total no. (%)	6/19 (31.6%)	9/19 (47.4%)
Non‐rebreathing mask – no./total no. (%)	0/19 (0.0%)	0/19 (0.0%)
Additional medication for COVID‐19 during trial		
Dexamethasone – no./total no. (%)	19/20 (95.0%)	20/20 (100.0%)
Remdesivir – no./total no. (%)	1/20 (5.0%)	1/20 (5.0%)
Tocilizumab – no./total no. (%)	0/20 (0.0%)	1/20 (5.0%)

Abbreviations: COVID‐19, Coronavirus disease 2019; SpO_2_, peripheral oxygen saturation.

^a^ This information was not available for one patient in both randomization groups.

### 3.2 Primary endpoint

**FIGURE 1:**
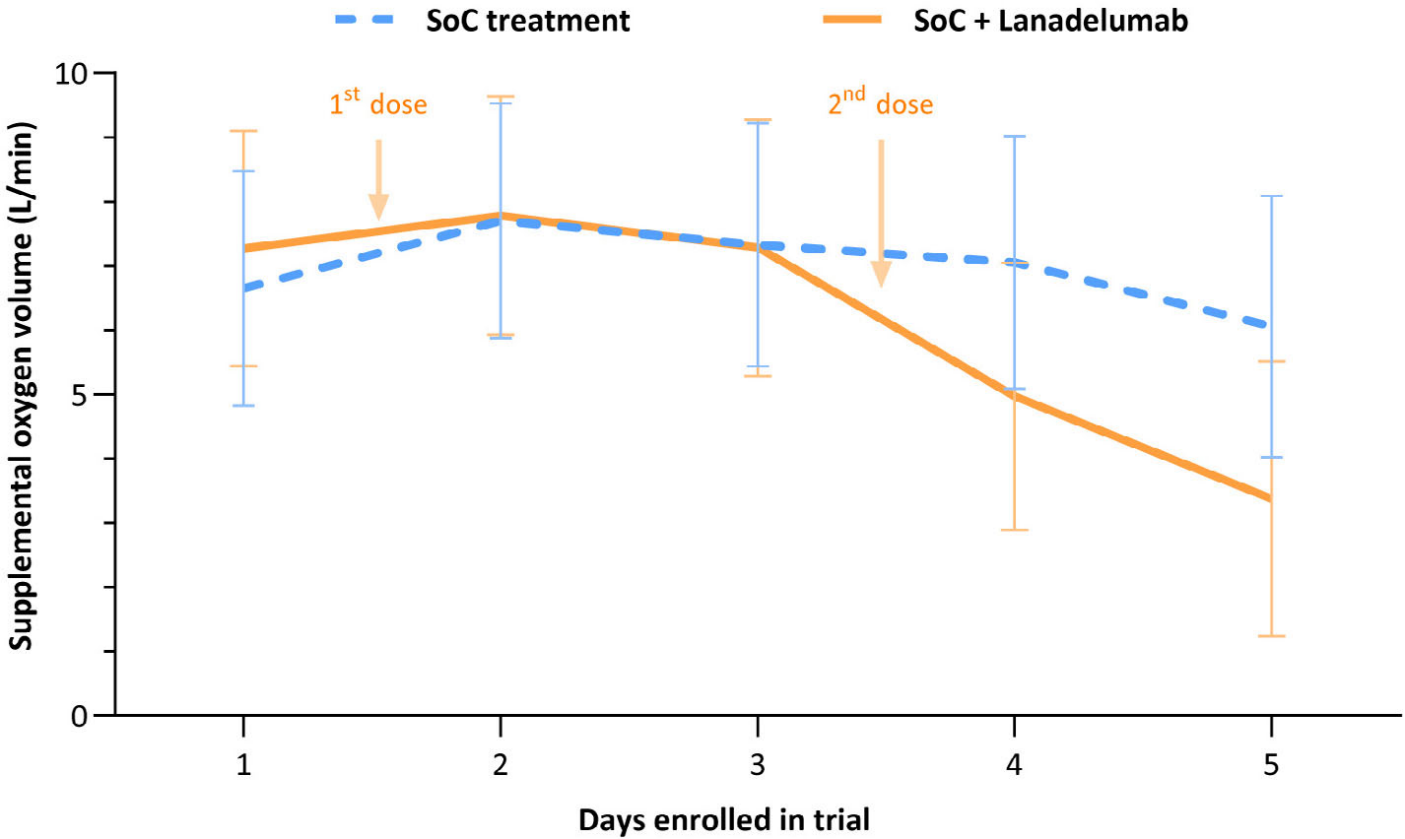
Predicted supplemental oxygen volumes during the first five trial days. Oxygen volumes were predicted with linear mixed model analysis, where treatment group, time and an interaction term of time x treatment group were entered as fixed effects. The continuous lines show the estimates of oxygen volumes per randomization group, and the error bars delineate the calculated 95% confidence intervals. Timing of the 1st and 2nd dose of lanadelumab administered to patients in the intervention group is indicated by the arrows at the top of the graph. Neither treatment group nor the interaction of treatment group and time significantly predicted supplemental oxygen volumes (p = 0.49 and p = 0.15, respectively).

Table 2 provides an overview of primary and secondary endpoints. At baseline, supplemental oxygen volumes ranged from 3 to 15 L/min in both groups, and there were no statistically significant differences in median supplemental oxygen between treatment groups (p = 0.43; Mann–Whitney U test). Figure 1 shows estimates of supplemental oxygen calculated by linear mixed model analysis. There was a substantial drop‐off in primary endpoint data, which was mainly due to treatment failure or hospital discharge. As a result of this, we were unable to reliably analyse the primary endpoint over the full 14‐day period as originally intended. Therefore, we decided to limit the analysis to the first five days of the trial, as at this point, data were still available from 14 patients in the control group and 13 in the intervention group, respectively. Within this timeframe, neither treatment group nor interaction between treatment group and time had a statistically significant effect on predicted supplemental oxygen (p = 0.49 and p = 0.15, respectively). When correcting for sex and Charlson Comorbidity Index (CCI), neither showed significant interaction with the treatment group. Correction for these factors did not alter our main conclusions (data on file).

**TABLE 2:** Summary of primary and secondary outcomes.

Primary outcome measure	Control group (N = 20)	Intervention group (N = 20)	p‐value
Mean supplemental oxygen volumes per day – first 5 days^a^			
Day 1 (L/min) – mean ± SD (N)	6.7 ± 2.9 (N = 20)	7.3 ± 3.5 (N = 20)	N.A.^b^
Day 2 (L/min) – mean ± SD (N)	7.7 ± 4.4 (N = 20)	7.6 ± 4.1 (N = 18)	N.A.^b^
Day 3 (L/min) – mean ± SD (N)	7.1 ± 5.0 (N = 16)	6.4 ± 4.1 (N = 13)	N.A.^b^
Day 4 (L/min) – mean ± SD (N)	6.4 ± 5.5 (N = 15)	4.2 ± 3.9 (N = 13)	N.A.^b^
Day 5 (L/min) – mean ± SD (N)	5.9 ± 5.0 (N = 14)	2.8 ± 2.7 (N = 13)	N.A.^b^
**Secondary outcome measures**			
Need for high‐flow oxygen therapy or mechanical ventilation			
High‐flow nasal cannula or non‐invasive ventilation – no./total (%)	6/20 (30.0%)	5/20 (25.0%)	1.0^c^
Mechanical ventilation – no./total (%)	2/20 (10.0%)	2/20 (10.0%)	1.0^c^
Hospital stay characteristics			
Duration of hospital stay in days – median (range)	8 (1–21)	6 (2–49)	0.81^d^
Need for ICU admission – no./total (%)	4/20 (20.0%)	5/20 (25.0%)	1.0^c^
Length of ICU stay in days – median (range)	7.5 (6–8)	6 (3–34)	0.91^d^
All‐cause mortality by day 14 – no./total (%)	1/20 (5.0%)	0/20 (0.0%)	1.0^c^
**Adverse events**			
All adverse events – no./total (%)	9/20 (45.0%)	7/18 (38.9%)	0.75^c^
Serious adverse events – no./total (%)	6/20 (30.0%)	5/19 (26.3%)	1.0^c^
Thromboembolic events – no./total (%)	4/20 (20%)	1/18 (5.6%)	0.34^c^
Liver test abnormality – no./total (%)	4/20 (20%)	3/18 (16.7%)	1.0^c^

Abbreviations: ICU, intensive care unit.

^a^ Mean oxygen volumes were calculated for patients using low‐flow supplemental oxygen. When treatment was escalated to high‐flow supplemental oxygen or mechanical ventilation, this was considered treatment failure and these data were not taken along in the primary outcome analysis.

^b^ These data were analysed using a linear mixed model (Figure 1).

^c^ Fisher's exact test.

^d^ Mann–Whitney U test.

To account for missing primary outcome data due to treatment failure or hospital discharge, we performed a sensitivity analysis using worst‐case imputation where patients receiving high‐flow oxygen or mechanical ventilation were assigned 15 L/min of oxygen, patients who had been discharged without supplemental oxygen were assigned 0 L/min, and patients who were discharged but still receiving oxygen were assigned 1 L/min. This allowed us to perform an analysis over the full 14 days. We did not detect a significant effect of lanadelumab on supplemental oxygen volumes at any timepoint. As KKS dysregulation likely occurs early in COVID‐19, we also performed subgroup analyses to detect whether timing of lanadelumab treatment may have influenced the primary outcome. While lanadelumab did tend towards efficacy over the first five days only in patients who were included at 10 days after onset symptoms or less, this trend was not observed over the full 14 days in this group using worst‐case imputation. The results of the sensitivity and subgroup analyses can be viewed in Supplementary File 3. Finally, we analysed the rates at which escalation to high‐flow oxygen was required. In the intervention group, five patients required high‐flow oxygen therapy via a high‐flow nasal cannula or non‐invasive ventilation mask, *vs*. six patients in the control group (25% and 30% respectively; p = 1.0, Fisher's exact test). Intubation and mechanical ventilation were necessary for two patients (10%) in both treatment groups.

### 3.3 Secondary endpoints

**FIGURE 2:**
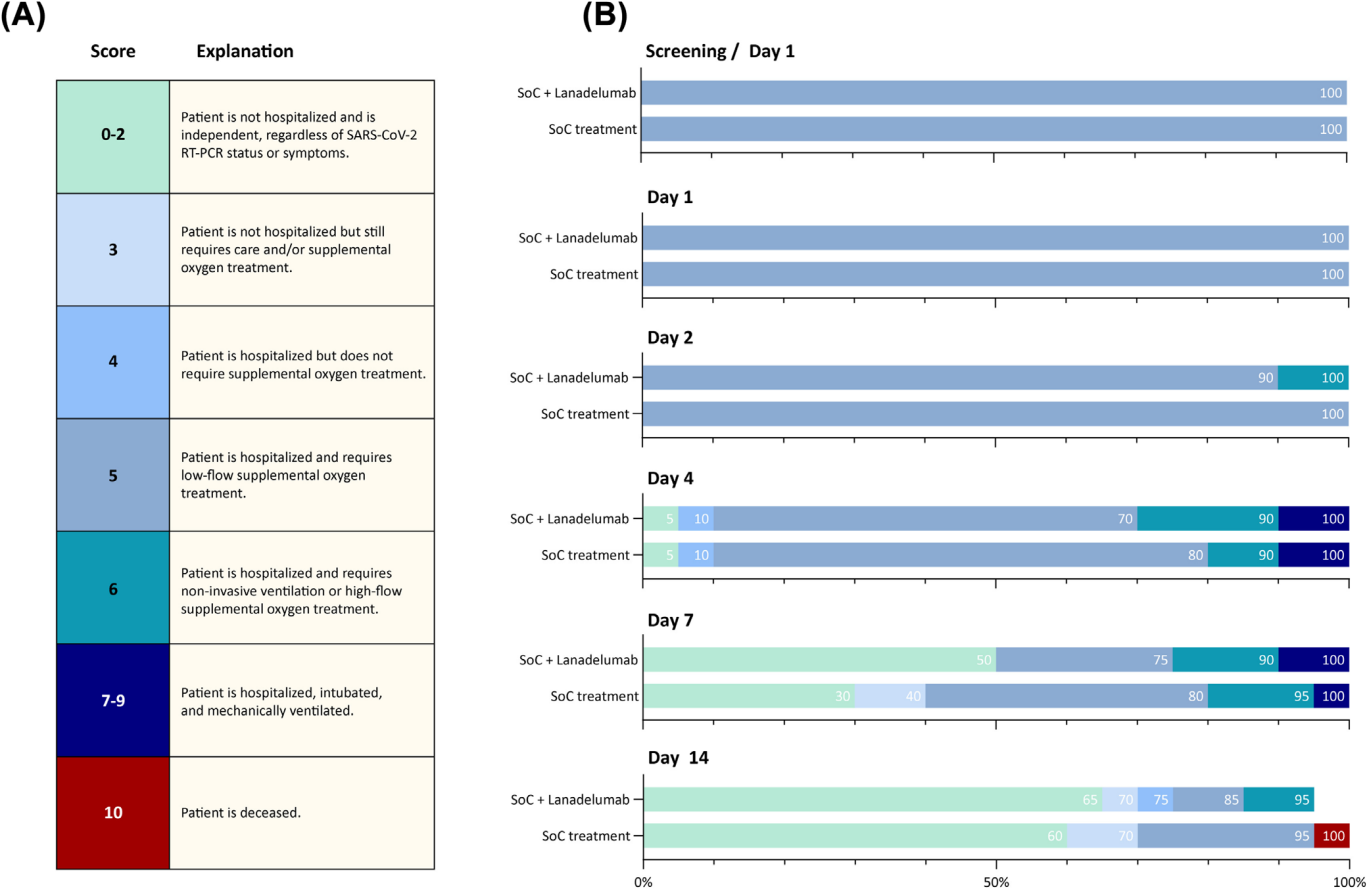
Modified WHO‐CPS scores and percentages of those scores in both treatment groups across key timepoints during the trial. (A) Modified WHO‐CPS scores and the clinical status corresponding to those scores. Scores indicated in the left column correspond with the original WHO‐CPS scores. Repeated PCR tests were not performed during this trial, so patients who were ambulatory and independent were scored as 0–2 regardless of PCR status or symptoms. Similarly, patients who required intubation and mechanical ventilation on the ICU were scored as 7–9. (B) Bar graphs indicating modified WHO‐CPS scores in each randomization group at key timepoints. The colours in the bars correspond to the colours of the modified WHO‐CPS scores indicated in Figure 2A. Percentages of patients in each randomization group are depicted in the bar segments. In the intervention group, modified WHO‐CPS data were missing for one patient due to loss to follow‐up.

In view of missing primary outcome data, we compared modified WHO‐CPS scores between both treatment groups. Modified WHO‐CPS scores were available for all timepoints in the trial, except for one patient in the intervention group who was lost to follow‐up from trial day nine onwards. At the time of enrolment, all patients were admitted to regular care wards and were treated with low‐flow supplemental oxygen, corresponding to a modified WHO‐CPS score of 5. Figure 2 shows the modified WHO‐CPS scale and the distribution of scores in both treatment groups at key timepoints.

Another secondary endpoint was the need for admission to the Intensive Care Unit (ICU), regardless of underlying reason. In the control group, four out of 20 patients (20%) required admission to the ICU, compared to five out of 20 in the intervention group (25%). This difference in ICU admission was not statistically significant (p = 1.0; Fisher's exact test). Median number of days spent in the ICU was 7 (range 3.0–8.0) in the control group compared to 6 (range 3.0–34.0) in the intervention group (p = 0.9; Mann–Whitney U test). Total duration of hospital stay was documented for all participants, allowing for evaluation beyond trial day 14. The median length of hospital stay was eight days in the control group compared to six in the intervention group (p = 0.91; Mann–Whitney U test). One patient in the control group died during follow‐up.

Pharmacokinetic (PK) data were available from a total of 17 patients in the intervention group. Observed data were collected following only a single dose for approximately 50% of patients. All patients were included in the PK analysis, with the exception of one in whom measurable concentrations on days 8 and 9 (12 905 and 10 172 ng/mL, respectively) were markedly lower than median values observed on those days (28 410 and 33 665 ng/mL, respectively). Median C_max_ (range) was 50.8 μg/mL (19.8–264). Other descriptive PK data can be found in Supplementary File 4.

### 3.4 Adverse events

Adverse events were documented for both treatment groups, and an overview is provided in Table 2. There was no significant difference in the reported number of adverse events between treatment groups (p = 0.8; Fisher's exact test). The most frequently reported adverse events were elevated biochemical liver tests (mainly alanine aminotransferase and aspartate aminotransferase), which occurred at similar rates between groups (p = 1.0; Fisher's exact test). Serious adverse events, including ICU admission, were also reported at similar rates (p = 1.0; Fisher's exact test). Thromboembolic events, which are a well‐known complication of COVID‐19, occurred in four patients in the control group compared to one in the intervention group (p = 0.32; Fisher's exact test).^37^, ^38^

## 4 DISCUSSION

This trial represents the first documented use of lanadelumab administered intravenously during active infection. In this small proof‐of‐concept study, we did not detect a potential benefit to lanadelumab treatment in addition to SoC on the primary outcome of supplemental oxygen volumes necessary to maintain SpO_2_ ≥ 93%. Similarly, lanadelumab did not significantly affect modified WHO‐CPS scores, need for admission to the ICU, total hospital stay duration, or 14‐day all‐cause mortality.

These results suggest less benefit to the use of lanadelumab compared to earlier observations of KKS inhibition in patients with COVID‐19 using the kinin B2 receptor blocker icatibant.^22^, ^23^ There are multiple plausible explanations for this. First of all, lanadelumab targets PKa, which is the enzyme responsible for the majority of bradykinin formation in plasma.^6^ Kinins produced by PKa may be less relevant to COVID‐19‐associated pulmonary oedema. Activity of lanadelumab at the site of injury (the pulmonary tissue and alveoli) could be limited due to its large structure as a monoclonal antibody.^19^ Kinins can also be generated through the action of tissue kallikreins, such as KLK1, which are expressed by a plethora of cell types.^39^, ^40^ Kinin generation by KLK1 is not affected by lanadelumab, whereas icatibant acts on B2R and can antagonize kinins generated by both PKa and KLK1.^18^, ^24^, ^41^ Timing of lanadelumab treatment could also affect its efficacy in combating COVID‐19‐associated pulmonary oedema. As KKS dysregulation is likely to be an early complication of SARS‐CoV‐2 infection, when viral particles are present at the site of injury in the lung, starting lanadelumab at a later stage would do little to counteract complex inflammatory responses caused by coagulation, complement and the innate and adaptive immune systems, let alone prevent a “bradykinin storm”. Finally, the effect of dexamethasone treatment is important to consider. Corticosteroids have been demonstrated not to affect bradykinin formation in human plasma directly, an observation underscored by their inefficacy in treating bradykinin‐mediated angioedema.^42^, ^43^ However, corticosteroids have been shown to inhibit the upregulation of bradykinin receptors in bronchial smooth muscle and airway epithelial cells induced by proinflammatory cytokines like IL‐1β.^44^, ^45^ Thus, dexamethasone may have dampened kinin‐mediated oedema in our study population indirectly, irrespective of lanadelumab treatment.

The similar rate at which adverse events occurred between treatment groups suggests a potentially beneficial safety profile for intravenous administration of lanadelumab, which is normally used subcutaneously. The most frequently reported adverse event was elevated biochemical liver tests, which are known to occur in approximately 4.7% of patients using subcutaneous lanadelumab for prevention of HAE attacks.^20^, ^21^, ^46^ In these patients, elevated liver enzymes are mostly transient and do not always warrant treatment cessation.^21^, ^46^ Liver enzyme elevation occurred in 16.7% of the intervention group and 20% of the control group, respectively. This rate of occurrence was greater than expected based on the literature and experiences with subcutaneous lanadelumab treatment in HAE patients. Taken together with the non‐significant difference in occurrence between treatment groups, this suggests that other causes, such as underlying disease, may explain these findings. Indeed, elevated liver enzymes have been described in up to half of all patients hospitalized with COVID‐19.^47^

This trial has several limitations. First, the exploratory nature and small sample size precluded any statistically meaningful conclusions on non‐inferiority or superiority of lanadelumab + SoC compared to SoC alone. The lack of blinding may have affected clinical decisions such as oxygen titration to achieve the target SpO_2_, assessment of clinical status and transfer to the ICU, as well as patient‐reported outcomes in terms of beneficial and adverse effects. This trial was conducted in an early phase of the pandemic, which limits its applicability to currently circulating SARS‐CoV‐2 strains. We were also unable to compare lanadelumab to other treatments used in COVID‐19, as all patients but one were also treated with dexamethasone, and only one was treated with tocilizumab. As we have shown, there were differences between the treatment groups at baseline, which is consistent with greater susceptibility to randomization imbalance in small trials. Both the mean age and the ratio of male to female participants were higher in the control group. These factors, combined with a higher median CCI, may have constituted a more disadvantageous risk profile for patients in the control group.^48^, ^49^ Although correcting for these differences in the primary endpoint analysis did not alter our conclusions, we cannot preclude that they may still have affected clinical outcomes and reduced statistical power to detect treatment effects. Furthermore, our modifications to the WHO‐CPS scores led to overestimation of scores for those in the 0–2 category and underestimation for those in the 7–9 category, resulting in less robust data. Finally, there are shortcomings inherent to using supplemental oxygen volumes as the primary outcome. As volumes used in high‐flow oxygen therapy and mechanical ventilation are not comparable to those used in low‐flow oxygen delivery systems, high‐flow oxygen volumes were excluded from evaluation. Earlier hospital discharge and use of oxygen therapy in home care similarly led to missing data points. These factors contributed to a considerable drop‐off in valid data points as the trial progressed, consequently providing an incomplete and possibly skewed representation of the primary outcome of the trial. For this reason, we chose to analyse the primary outcome over the first five days after trial enrolment, as after this timepoint, oxygen data were available for less than half of the study participants. This limited our assessment of the efficacy of lanadelumab after the first five trial days and precluded the detection of delayed treatment effects. It also remains uncertain whether lanadelumab treatment could have been beneficial at earlier or less severe stages of COVID‐19, and the appropriate window for treatment may have been missed. In participants enrolled within 10 days of symptom onset, lanadelumab tended towards efficacy only over the first five trial days. However, the results of this subgroup analysis should be interpreted with caution as statistical power was limited by small sample size (n = 23) and truncated endpoint data. Future studies may benefit from using composite outcomes such as WHO‐CPS scores instead of supplemental oxygen volumes as the primary endpoint. This would allow for more comprehensive and uniform assessment of treatment efficacy across diverse patient populations and treatment contexts over a longer period of time.

## 5 CONCLUSION

We conducted an exploratory interventional study investigating the potential efficacy and safety of intravenous lanadelumab in moderately ill hospitalized COVID‐19 patients. Though hypothesis‐generating in nature, our findings do not suggest a beneficial effect of lanadelumab in addition to SoC treatment on the primary outcome of supplemental oxygen need during the first five trial days, nor the secondary endpoints of modified WHO‐CPS scores over the course of the trial, need for high‐flow oxygen therapy or mechanical ventilation, admission to the ICU, length of hospital stay, or 14‐day all‐cause mortality. Limitations such as small sample size, drop‐off in primary endpoint data and randomization imbalances call for restraint in the interpretation of our results. Although intravenous lanadelumab did not affect clinical outcomes, it was well tolerated in our study population. The similar occurrence of adverse events between groups suggests a favourable safety profile of intravenously administered lanadelumab during active infection. Considering the promising results reported when using icatibant in early COVID‐19, downstream KKS inhibition may yet remain an interesting treatment option for bradykinin‐mediated pulmonary oedema in COVID‐19. Moreover, analyses of the coagulation system, cytokine responses and the KKS from patients with or without blocked PKa activity in vivo may provide more biological insights into the pathogenesis of COVID‐19 and the involvement of the KKS. A manuscript concerning the pharmacokinetic and pharmacodynamic data collected as part of this trial is currently in preparation.

## AUTHOR CONTRIBUTIONS

JE, FvdV, RB designed research; JE, RJH, CL, HL, RS, WJW performed the research and recruited the patients; JE, FvdV, RB, KR, CM analysed the data; JE, FvdV and RB wrote manuscript; all others provided critical revision of the manuscript. All authors provided approval of the final version of the manuscript.

## CONFLICT OF INTEREST STATEMENT

No conflicts of interest or competing interests are applicable to this work.

## Supporting information


**Data S1.** Supporting Information.

**Data S2.** Supporting Information.

**Data S3.** Supporting Information.

**Data S4.** Supporting Information.

## Data Availability

The data presented in this manuscript has been stored at Radboudumc, Nijmegen, the Netherlands and can be made available upon request to the corresponding author.
