# Supplementary material for: Intravenous lanadelumab for the treatment of moderately ill COVID‐19 patients
Source: Br J Clin Pharmacol. 2026 Jan 9;92(6):1685–95. doi: 10.1002/bcp.70438 (PMC13206366; doi:10.1002/bcp.70438)
Supplement: Supplementary file 1 — Data S1. Supporting Information. [file BCP-92-1685-s004.pdf]

**Study Title**:	Lanadelumab for treatment of COVID-19 disease
**Protocol ID**:	UMCN-AKF-20.04
**Acronym**:	Covid-lanadelumab
**EudraCT number**:	2020-002472-12
**ABR number**:	NL74095.091.20
**Sponsor:**	Radboud University Medical Center<br>Geert Grooteplein 10<br>6525 GA Nijmegen, The Netherlands
**Principal Investigator/ conductor**	Dr. R.J.M. Brüggemann<br>Radboud University Medical Center<br>Dept. of Pharmacy<br>Geert Grooteplein 10<br>6525 GA Nijmegen, The Netherlands
**Medical Investigator**	Dr. F.L. van de Veerdonk (co-Pi)<br>Radboud University Medical Center<br>Dept. of Internal Medicine, Radboudumc, Nijmegen<br>Geert Grooteplein 10<br>6525 GA Nijmegen, The Netherlands
**Independent expert Monitor:**	Dr. Monique Reijers, longarts<br>Radboud University Nijmegen Medical Center
**Co-PI:**	Dr. I. Kouijzer, Department of Internal Medicine, Radboudumc, Nijmegen<br>Dr. L. Derde, Department of Intensive Care, UMC Utrecht, Utrecht<br>Dr. R. ter Heine, Department of Pharmacy Radboudumc, Nijmegen
**Statistician:**	Prof. Kit Roes, Radboud University Nijmegen Medical Center
**Subsidising party**	Radboudumc, department
**Laboratory**	Laboratory of the Department of Pharmacy, Radboud university medical centre, Geert Grooteplein-Zuid 10, 6525 GA, Nijmegen, The Netherlands<br>864 Radboud university medical centre
**Pharmacy**	Clinical Trials Unit of the Department of Pharmacy, Radboud University Medical Centre, Geert Grooteplein-Zuid 10, 6525 GA, Nijmegen, The Netherlands<br>864 Radboud university medical centre
**Project Manager:**	Dr. Roger JM Brüggemann<br>Dept. of Pharmacy<br>864 Radboud University Medical Center<br>Geert Grooteplein 10<br>6525 GA Nijmegen, The Netherlands<br>Telephone: (+31) 24 3616405<br>Fax: (+31) 24 3668755
**Subsidizing parties**	ZONMW Projectnummer 50-56300-98-133 : A phase-2-study, pivotal for clinical development of lanadelumab for treatment of COVID-19
**Protocol Version/Date:**	15-DEC-2020

TRIAL ACKNOWLEDGEMENT

Lanadelumab for treatment of COVID-19 disease

Protocol UMCN-AKF 20.04– version 11 January 2021

INVESTIGATOR STATEMENT

I have read the protocol, including all appendices, and I agree that it contains all necessary details for me and my staff to conduct this study as described. I will conduct this study as outlined herein and will make a reasonable effort to complete the study within the time designated.
I will provide all study personnel under my supervision copies of the protocol. I will discuss this material with them to ensure that they are fully informed about the drugs and the study.

Sponsor / principal investigator

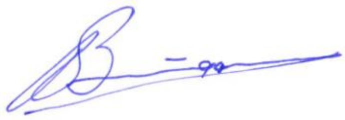

____

Dr. Roger Brüggemann (Printed) Signature

11-01-2021

____

Date

PROTOCOL SYNOPSIS

**GENERAL INFORMATION**	
Study title	Lanadelumab for treatment of COVID-19 disease
Trial number	UMCN-AKF 20.04
Start Date	October 2020
Duration of Study	12 months
Clinical phase	NA
Sponsor	Dept. of Pharmacy, Radboud University Medical Center (Radboudumc), Nijmegen, The Netherlands
Co-ordinating Investigator (principal investigator)	Dr. R.J.M. Brüggemann, Dept. of Pharmacy, Radboudumc, Nijmegen, The Netherlands
Investigator (medical)	Dr. F.L van de Veerdonk, Dept of Internal Medicine, Radboudumc, Nijmegen, The Netherlands
Participating hospitals	• Amsterdam UMC, Amsterdam<br>• Radboud University Medical center, Nijmegen,<br>• University Medical Center Utrecht, Utrecht,<br>• Rijnstate Hospital, Arnhem<br>• Flevoziekenhuis, Almere
Experimental design	Open-lable, randomized controlled, intervention, multi-center study
Objective(s)	The primary objectives of this study is as follows:<br>• To generate the proof of concept for lanadelumab for treatment of symptoms moderate to severe patients COVID-19 disease<br>• To demonstrate its safety after intravenous administration.
Rationale	So far little is very few drugs have demonstrated positive results for treatment of COVID19.<br><br>Recently we have shown that the use of icatibant in COVID-19 results in a potent decrease in oxygen use. Yet the effect of the three dosages as according to the label dose was insufficient to maintain the clinical improvement in a small group of patients. We argue that with the use of lanadelumab a more lasting effect can be reached due to its longer half life.
Study subjects	Patients tested positive for COVID-19 (PCR), that are admitted to the general ward. Fourty patients will be enrolled in a 1:1 randomized fashion for intervention (lanadelumab administration) versus controls (standard treatment). In addition, 40 historic controls (COVID positive without lanadelumab matched for the 20 patients that have received lanadelumab) will be included.<br>Randomization will be done by Castor.<br><br>The sample size is empirical as this is a hypothesis-generating study.<br><br>Inclusion criteria:<br>1. Patient is SARS-COV2 positive (PCR)<br>2. Without oxygen a saturation below 90%And / Or At least 3L/min oxygen dependent<br>3. Patient is 16 years and older<br><br>Exclusion criteria:<br>1. Has previously participated in this study<br>2. Acute myocardial or cerebral ischemic event at time of enrolment

	3. Receiving ACE or ARB inhibitor or comparable drugs that is specified as an intervention in this domain as a usual medication prior to this hospitalization will exclude a patient from receiving that agent<br>4. A baseline alanine aminotransferase or an aspartate aminotransferase that is more than five times the upper limit of normal<br>5. Patient is known hypersensitive to full human monoclonal antibodies<br>6. Patient is pregnant or breast feeding

**TREATMENTS**	
Treatments	For the intervention patients will receive an intravenous dose of 300 mg lanadelumab on day 1, followed by a second dose of lanadelumab 300mg iv on day 4. The other 20 lanadelumab patients will receive the same treatment algorithm while NOT receiving lanadelumab.
**ASSESSMENTS**	
• Co medication	All co-medication will be registered during study period.
**ANALYTICAL AND STATISTICAL METHODS**	
Safety, demographics and statistics	In general, all subjects who completed the study will be included in the statistical evaluation for demographics and safety. Subjects can be excluded from the statistical analysis if no reliable parameters can be determined. Moreover, subjects can be excluded from data evaluation if justified by circumstances (e.g. no correct drug administration). Patients are regarded evaluable after administration of the first lanadelumab dose.<br><br>Statistical analysis will be carried out using SPSS software version 20 [SPSS Inc.]. Descriptive statistics will be calculated using Excel software [Microsoft Corporation].
Risk analysis	The risk-classification is assessed as low / moderate to the study population receiving study drug at the current regimen. Lanadelumab has a favorable safety profile and is approved by the Dutch medicines evaluation board for the dose administered. Therefore the risk for adverse events is low.
Nature and extent of the burden and risks associated with participation, benefit and group relatedness	The study participants are COVID-19 patients that might benefit from the participation in this clinical trial.<br><br>Participants are on the ward of the time of study<br><br>Burden: short visits (15 minutes) several time per day on the ward by doctor and nurse for clinical evaluation. The duration of the entire trial (excluding screening period) is 14 days. Duration of treatment with study medication is 2 days.<br><br>Lanadelumab has a generally favorable safety profile. We refer to the SmPC for most common side effect.<br><br>For pharmacokinetic purposes 14 blood samples will be taken in total in the intervention group only. For a total of 28 mL. Additional samples will be taken for biomarker evaluation (two occasions of 40mL each).<br><br>Safety assessment (haematology, clinical chemistry, blood glucose tests, hCG blood and hCG urine tests, serology) will be collected as part of routine care. The total blood volume taken will be approximately 100 mL..

ANALYTICAL AND STATISTICAL METHODS	
Bioanalysis	Plasma concentrations of lanadelumab will be performed via Takeda trough the commercial lab that has performed sample measurements in their clinical trial.
Pharmacokinetics	Non-linear mixed effect modelling will be deployed by the department of Pharmacy and the Radboud Applied Pharmacometrics group.
Safety and demographics	Tabulation and descriptive statistics for subject characteristics. Tabulation of adverse events, vital signs and biochemistry and haematology data.<br><br>Statistical evaluation will be supervised by Prof. Kit Roes.

Table of contents

1. INTRODUCTION 8
1.1 Background 8
1.1.1. COVID-19 infection 8
1.1.2. ACE2 as the 'entry' receptor for SARS-CoV-2 infection 8
1.1.3. ACE2-deficiency and kinin-kallikrein activation 8
1.1.4. Potential strategies to block ACE2-deficiency-mediated kinin-kallikrein activation. 9
1.2 Rationale for the current study 10
1.3 Primary objectives 15
2. STUDY DESIGN 16
3. SUBJECT POPULATION 17
3.1 Number of subjects and subject selection 17
3.2 Inclusion criteria 17
3.3 Exclusion criteria 17
3.4 Study duration 17
3.5 Removal of subjects from the study 17
3.6 Replacement of subjects 18
3.7 Stopping rules for the study 18
4. TREATMENT 19
4.1 Dose rationale and rate of infusion 19
4.2 Drug interactions 19
4.3 Safety and potential risks 19
5. TRIAL DRUGS 22
5.1 Identity of investigational product(s) 22
5.2 Packaging and labelling of trial medication 22
5.3 Method of assigning subjects to treatment groups 22
5.4 Selection and timing of dosing and dietary 23
5.5 Treatment Compliance 23
5.6 Drug accountability 23
5.7 Prior and Concomitant Therapy 23
6. STUDY PROCEDURES 24
6.1 Inclusion Screening and screening during treatment 24
6.2 Safety assessments 25
6.3 Safety reporting 25
7. DATA ANALYSIS 29
7.1 Sampling design and sample size justification 29
7.2 Data collection and data management 29
7.3 Statistical analysis 29
8. STRUCTURED RISK ANALYSIS 30
9. ETHICAL CONSIDERATIONS / RESPONSIBILITIES 31
9.1 Investigator Responsibilities 31
9.1.1 Good Clinical Practice 31
9.1.2 Institutional Review Board (IRB)/Independent Ethics Committee (IEC) Approval 31
9.1.3 Informed Consent 31
9.1.4 Benefits and risks assessment, group relatedness 31
9.1.5 Incentives 32
9.1.6 Confidentiality 32
9.1.7 Study Files and Retention of Records and Samples 32
9.1.8 Case Report Forms 33
9.1.9 Inspections 33
9.1.10 Protocol Compliance 33
9.1.11 Insurance Cover 33
9.2 Sponsor Responsibilities 33

9.2.1 Protocol Modifications 33
9.2.2 Study Progress report 33
9.2.3 Study Report and Publications 33
9.3 Joint Investigator/Sponsor Responsibilities 34
9.3.1 Access to Information for Monitoring 34
9.3.2 Study Discontinuation 34
9.4 Facilities 34
10. ADMINISTRATIVE ASPECTS, MONITORING AND PUBLICATION 35
10.1 Handling and storage of data and documents 35
10.2 Monitoring and Quality Assurance 35
10.3 Amendments 35
10.4 Annual progress report 36
10.5 End of study report 36
10.6 Public disclosure and publication policy 36
11. REFERENCE LIST 37

1. Introduction

1.1 Background

1.1.1. *COVID-19 infection*

The first report of infection with COVID-19 occurred in Wuhan, China, in late 2019. Since that time, , there have been multiple millions of reported cases across the world with a range of severity, several thousand deaths and documented sustained human-human transmission. On January 30th 2020, the World Health Organization (WHO) declared this outbreak a Public Health Emergency of International Concern. Given past history with novel coronaviruses, such as Severe Acute Respiratory Syndrome (SARS) and Middle East respiratory syndrome coronavirus (MERS-CoV), public health agencies have responded aggressively to the urgent need to acquire knowledge regarding this emerging infection. An important component of this urgently needed knowledge includes understanding the effectiveness of alternative treatment strategies in patients with suspected or proven infection.

Estimates of the burden of critical illness among patients infected with COVID-19 vary, with estimates of case-fatality and proportion of patients who become critically ill being unstable. Several factors contribute to this uncertainty including differential timing between diagnosis and development of critical illness or death, the true incidence of infection being uncertain because of possible under-reporting of asymptomatic or mild cases, the sensitivity of diagnostic methods, possible limitation on the number of diagnostic tests that can be performed, and changing case-definitions.

1.1.2. *ACE2 as the 'entry' receptor for SARS-CoV-2 infection*

Receptor recognition is the first step of a viral infection and is a key determinant of host cell and tissue tropism. The metallopeptidase angiotensin-converting enzyme 2 (ACE2) has been identified as the functional entry receptor for SARS-CoV-2, similar to SARS-CoV (Walls et al., 2020). ACE2 is expressed on lung alveolar epithelial cells and enterocytes of the small intestine and it is present in arterial and venous endothelial cells and arterial smooth muscle cells (Hamming et al., 2004). SARS-CoV-2 has a Spike protein that harbors a furin cleavage site at the S1/S2 boundary, which can be processed by the host cellular protease TMPRSS2, in turn modulating the Spike protein such that it can then bind to ACE2, allowing the SARS-CoV-2 virus to enter the cell (Hoffmann et al., 2020; Walls et al., 2020).

The fact that SARS-CoV-2 binds with ACE2 receptors might explain why COVID-19 pathology is predominantly a disease of the alveoli - where ACE2 is widely expressed (Hamming et al., 2004; Li et al., 2020) – often resulting in acute respiratory distress syndrome (ARDS). The virus-induces a strong proinflammatory response that results in endothelitis which affects multiple organs (Varga et al., 2020) and results in vascular and thrombotic complications such as vascular leakage and micro-thrombosis. Similar to the pathological lung changes observed with SARS, these potentially ACE2-mediated pathophysiological features are already present at an early disease stage within the first 10 days (Gu and Korteweg, 2007) and provide a rationale to target ACE2 entry and interaction with the virus early.

1.1.3. *ACE2-deficiency and kinin-kallikrein activation*

With respect to the kinin-kallikrein system there is a clear role for ACE2. Bradykinin (BK) is a linear nonapeptide that is formed by the proteolytic activity of kallikrein on kininogens (Bhoola et al., 1992). Kallikreins are serine proteases and can be divided in plasma kallikrein and tissue kallikreins. The plasma and tissue kallikreins release the vasoactive peptides known as kinins, which cause relaxation of vascular smooth muscle and increased vascular permeability (Bhoola et al., 1992; Marceau et al., 2018). Plasma kallikrein processes high-molecular-weight kininogen (HMWK produced by the liver) into bradykinin, while tissue kallikrein processes low-molecular-weight

kininogen (LMWK produced by the liver) and results in Lys-BK (29802875). These are the ligands for the constitutively expressed bradykinin receptor B2 on endothelial cells. In addition, the enzymes (carboxypeptidase M (CPM) and carboxypeptidase N (CPN)) can further process BK and Lys-BK into des-Arg9-BK and Lys- des-Arg9-BK respectively, which are ligands for bradykinin receptor B1, a receptor on endothelial cells that is upregulated under proinflammatory conditions (Marceau et al., 2018). These kinins have strong vasopermeable and vasodilatory capacity and are tightly controlled to prevent excessive angioedema (Marceau et al., 2020). ACE and ACE2 both have roles in inactivating the ligands for the bradykinin receptors (Gralinski et al., 2018). ACE mainly inactivates bradykinin which is the major ligand for B2 receptors, while ACE2 does not inactivate bradykinin, but can inactivate des-Arg9-BK, which is a potent ligand of the B1 receptor in the lung and can be upregulated by inflammation (Hess et al., 2017; Sodhi et al., 2017). In this way, ACE2 can be protective against pulmonary edema, especially in the setting of inflammation(Imai et al., 2005). Therefore, ACE2 deficiency could lead to impaired inactivation of the B1 ligands locally in the lung, triggering a cascade that leads to vascular leakage, activation of zymogen systems such as plasma kallikrein, promoting B2 signaling and activation of the contact system [figure 1]. Such a cascade would lead to elevation of D-dimers and microthrombosis, both of which are commonly reported in COVID-19 disease (Fox et al., 2020; Poor et al., 2020; Xu et al., 2020).

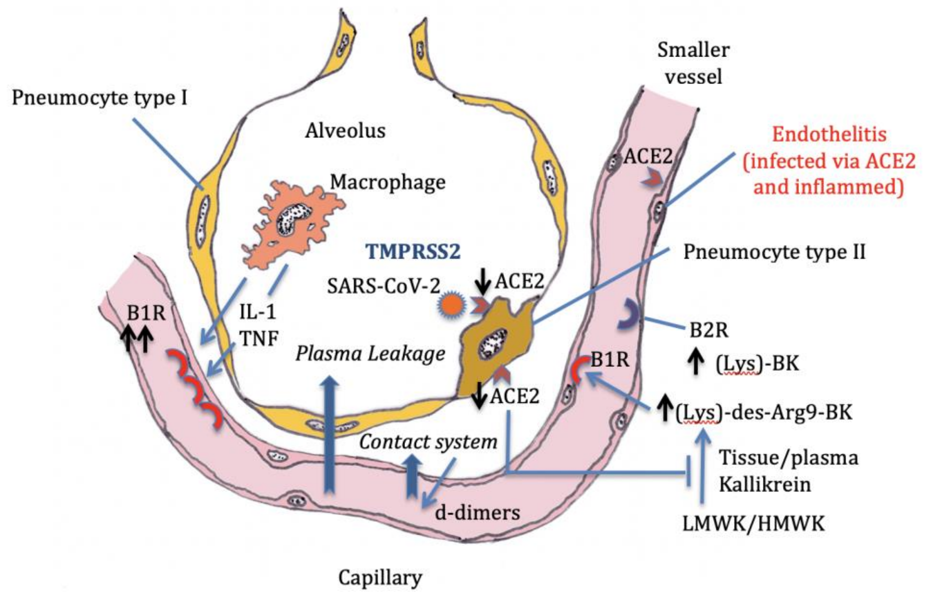

Figure 1. Conceptual model of SARS-CoV-2 interaction with ACE2 receptor and proximate downstream signaling. SARS-CoV-2 reaches the alveolus. The spike protein is transformed by the host serine protease TMPRSS2, allowing the virus to bind to ACE2. ACE2 expression is downregulated resulting in ACE2 deficiency locally. This will result in the incapacity to neutralize B1 ligands, especially des-Arg9-BK leading to plasma leakage. Inflammation will increase the expression of Bradykinin 1 receptor (B1R), increasing the plasma leakage. Plasma leakage will then contribute to B2R activation, maintaining vascular leakage and activating the contact system in the subendothelial space, with d-dimer leakage into the circulation. The virus has now the option to infect local endothelial cells, causing endothelitis, which can continue into the larger blood vessels. An uncontrolled continuation of this status will result in microthrombosis and eventually in fibrosis.

1.1.4. *Potential strategies to block ACE2-deficiency-mediated kinin-kallikrein activation.*

The kinin-kallikrein system could be explored as a possible treatment target. Blocking the bradykinin receptors (B1R and B2R) would be a strategy. However, there are currently no available B1R blockers. Two drugs are commercially available to target these symptoms. A B2R blocker, icatibant (Firazyr®), is approved for the treatment of patients with hereditary angioedema (HAE) and ACE inhibitor

induced angioedema (Dubois and Cohen, 2010). And an anti-kallikraine monoclonal antibody lanadelumab that is approved for the prevention of HAE. Given the abundance of the plasma leakage resulting in activation of B2 receptors at the site of infection (Imamura et al., 2005; Sodhi et al., 2017; Taylor et al., 2013), there is a sound scientific rationale to explore lanadelumab as immune modulatory strategy in patients with moderate and severe COVID-19 disease.

1.2 Rationale for the current study

In a proof-of-concept study in patients with COVID19 disease we have demonstrated the involvement of the kinin-kallikrain system.

To support the rationale to target the kallikrein-kinin system in COVID-19, we investigated whether we could identify active plasma kallikrein (PKa) in the plasma of patients. Plasma prekallikrein (PK) needs to be cleaved into its active form that in turn will result in formation of bradykinin. All patients with severe COVID-19 showed a lower concentration of uncleaved PK, with a concomitant significant higher concentration of PKa compared to healthy volunteers. All patients with COVID-19 had a significant increased PKa compared to healthy controls (Figure 2). These data support the concept of an increased activation of the plasma kallikrein-kinin system in patients with COVID-19.

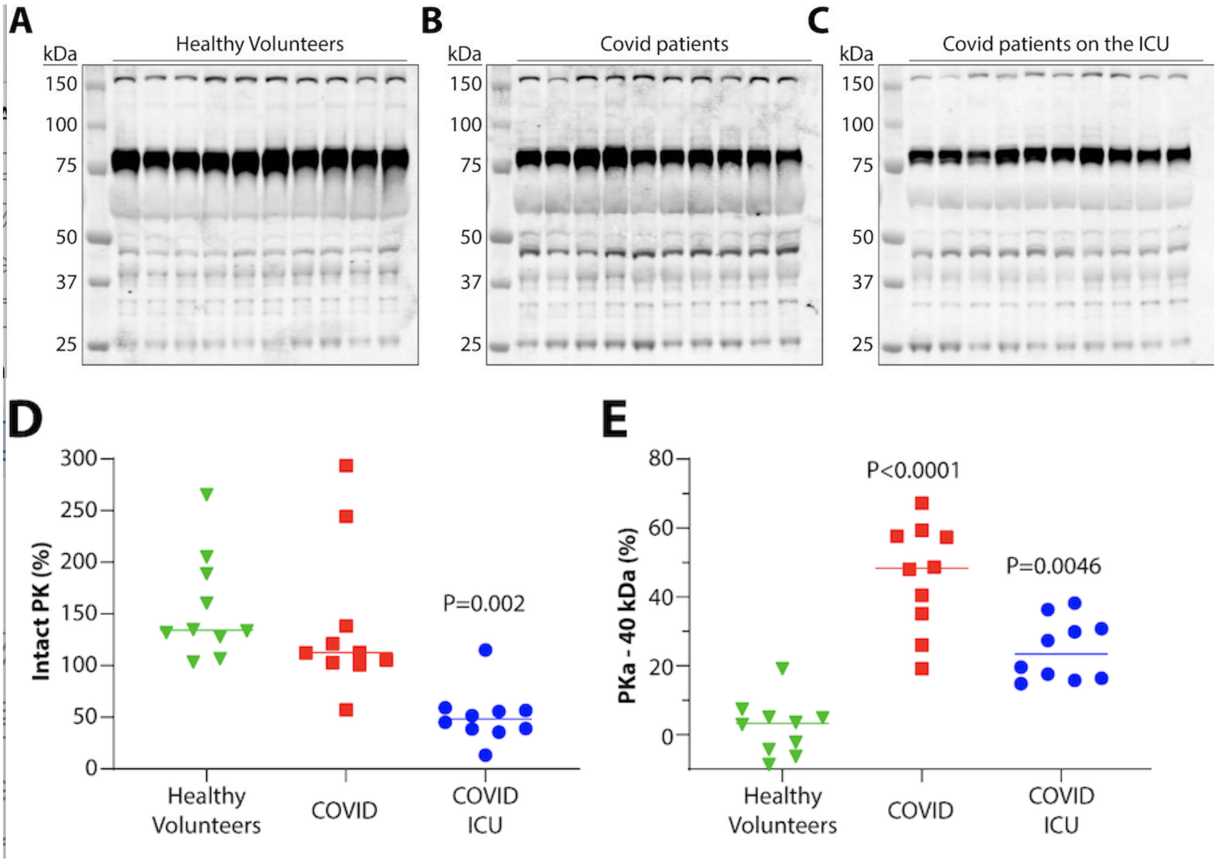

Figure 2: Prekallikrein (PK) and kallikrein active fragments (PKa) in plasma of 20 patients with COVID-19 and ten healthy volunteers. (A). Western blots showing prekallikrein (PK) and active plasma kallikrein fragments (PKa) in the plasma of healty volunteers, (B) 10 patients with COVID-19 admitted on the ward, and (C) 10 patients with COVID-19 admitted to the ICU. (D). Intact PK and (E) PKa fragments quantified by using dextran activated samples of a normal pool plasma to create a standard curve. EDTA-plasma samples from healthy volunteers and COVID-

patients were related to this standard via linear regression. Statistics were performed by one-way ANOVA with Dunnett multiple comparison test. P<0.05 was considered significant.

<u>Effect of icatibant on oxygen need</u>

To provide proof of concept that the plasma kallikrein-kinin system could be a therapeutic target in COVID-19 to reduce local pulmonary angioedema and subsequently improve oxygenation in the lungs, we administered icatibant in 10 patients with COVID-19. Oxygen status of all patients and three matched controls per patient are shown in Figure 3 with the exception of patient 3.

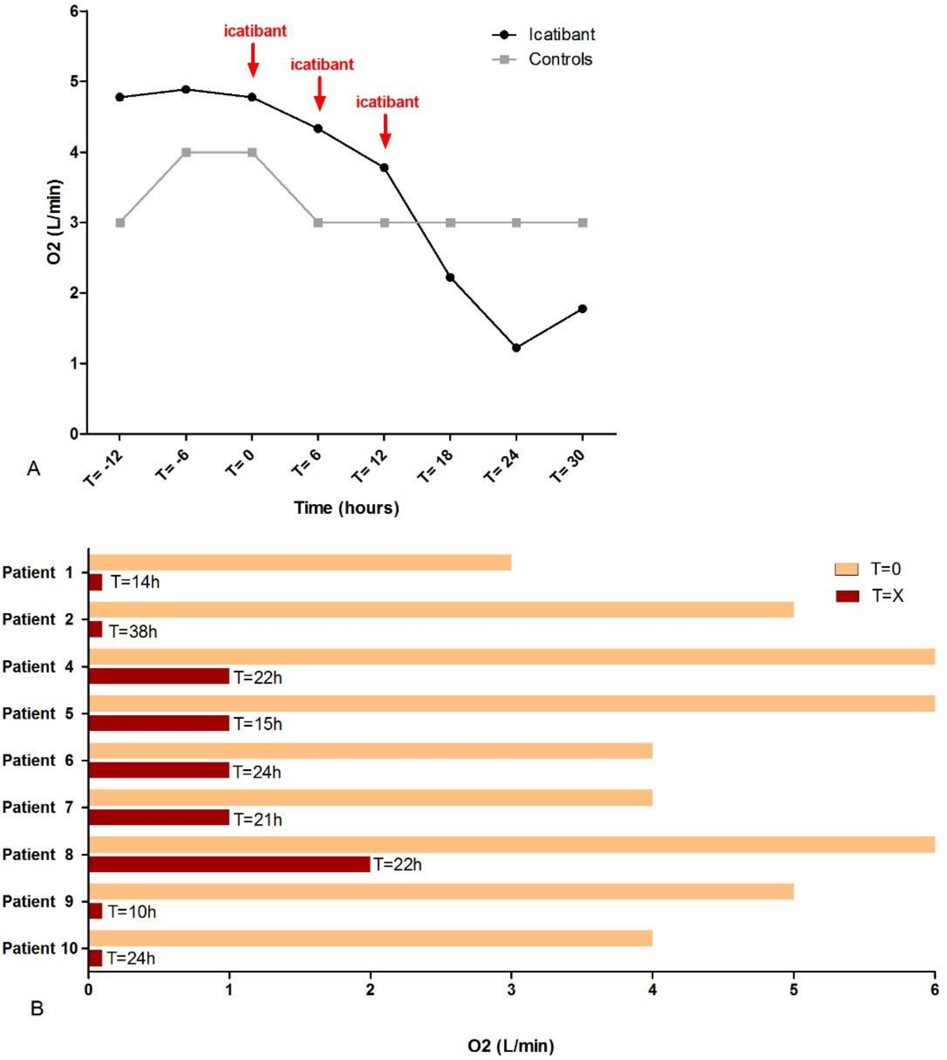

Figure 3. Oxygen supplementation in COVID-19 patients treated with icatibant versus controls. A. Median oxygen supplementation in COVID-19 patients with icatibant treatment (n=9) versus COVID-19 patients without icatibant treatment (n=27). T=0 is start icatibant (patients) or 24 hours after admission (controls). B. Reduction in oxygen supplementation in individual patients (n=9).

This patient started with icatibant in the ICU while on high-flow nasal oxygen (Optiflow). It was difficult to assess the effect of icatibant in this patient. Nine patients started with icatibant on the ward. In all 9 patients there was a marked decrease in oxygen supplementation (Figure 2). After

three injections of icatibant 4 patients were no longer oxygen dependent within 10-35 hours. In 5 patients there was a substantial decrease of oxygen supplementation after treatment with icatibant (Figure 3). Overall, in 8 out of 9 (89%) patients treated with icatibant a reduction of ≥ 3L/min in oxygen supplementation after 24 hours was observed (Figure 3). Out of 27 controls 3 (11%) showed a spontaneous reduction in oxygen supplementation of ≥ 3L/min within 24 hours. We noted that in 3 patients treated with icatibant there was a resurgence in the need for oxygen supplementation (Figure 2). A rapid reduction in oxygen need followed by a resurgence in the oxygen need was not observed in the matched controls with COVID-19

Effects of icatibant on other clinical end-points

Eight of the 10 patients noticed clinical improvement within hours after administration of icatibant. These subjective findings varied from 'less difficulty in breathing', to 'easier cough', to simply 'just feeling better'. In 9 out of 10 patients, no ICU admissions were needed and patients remained on the COVID ward after treatment with icatibant. In one patient (patient 7), respiratory status deteriorated 5 days after administration of icatibant. This patient was transferred to the ICU and needed invasive mechanical ventilation. For all 10 patients there was no clear effect on D-dimer concentrations during the first 24 hours after the start of icatibant treatment. Other laboratory parameters and fever did also not differ from the matched controls.

Based upon these results we feel that this opens up two aspects.

1) To move from icatibant with a very short half-life (around 2 hours) towards lanadelumab (half life about 14 days)
2) To administer lanadelumab intravenously as a one-hour infusion to safeguard immediate effect on free plasma kallikraine.

Ad 1. Clinical rationale to support the first aspect: As icatibant has a short t1/2 of approximately 1.4 hours after a single subcutaneous 30mg dose, a medication with a significantly longer half-life that could prevent the production of bradykinin in the first place may impart additional therapeutic benefit. As described in greater detail in subsequent sections, lanadelumab is a fully human monoclonal antibody (IgG1/κ-light chain) that binds plasma kallikrein and inhibits its proteolytic activity to reduce the production of bradykinin. It is currently indicated in the United States for prophylaxis to prevent attacks of hereditary angioedema (HAE) in patients 12 years and older. In contrast to icatibant, lanadelumab has longer t1/2 of approximately 15 days. The advantage in reducing BK production is that not only would this result in lower activity at BKR2, but since levels of its metabolite desArg9-BK would similarly be reduced, would also result in lower activity at BKR1.

Additionally, it is reasonable to hypothesize that plasma kallikrein inhibition may help to reduce inflammation and coagulation driven by FXII which is activated by plasma kallikrein through a positive feedback loop. However, this is still speculative and awaits scientific validation.

Therefore, lanadelumab, given its mechanism of action coupled with the available literature, and anecdotal reports of icatibant use in COVID-19, provides a reasonable rationale for the study proposed which seeks to evaluate the efficacy and safety of lanadelumab to reduce/prevent acute respiratory distress syndrome associated with COVID-19 pneumonia.

Ad 2. In the registration package, PK/PD properties of lanadelumab have been well characterized in healthy volunteers and HAE patients (aged between 12 to 75 years old), following 300 mg q2w SC administration (approved dosage for HAE indication). The characterization conducted using a

population PK/PD modeling and simulation approach showed that the 300 mg q2w SC with approximately 15 days half-life provides sufficient exposure for HAE efficacy with well-demonstrated safety profiles. Following SC administration of 300 mg q2w, the Cmax was approximately 34 ug/mL and the Tmax was 4 days or longer, which may not be sufficient to meet the early treatment needs (regarding the rate and extent of SC absorption) in patients with progressing disease of COVID-19 pneumonia. Rapid progression of pulmonary deterioration from early clinical symptoms to severe in patients admitted into the hospital clearly demonstrates the need for rapid, high, and safe exposure of effective medicines. IV administration of lanadelumab is expected to meet such a need and 300 mg IV will provide much earlier Tmax and higher concentrations in the earlier PK phase compared to 300 mg SC administration. Although the sponsor has not tested the IV route of administration in the HAE clinical package, the exposure to lanadelumab following an IV administration of 300 mg (predicted Cmax: 94.1 ug/mL) assuming 66% SC bioavailability) is expected to be comparable to observed exposure in the clinical HAE package, and the nonclinical package. Therefore, the sponsor selects IV administration for the proposed clinical pharmacology study.

In the pop-PKPD document [attached as supplemental file] extensive information is provided why the intravenous administration is preferred over the subcutaneous administration.

Finally, in the next paragraphs information on safety of lanadelumab will be outlined further supporting the need for this drug in COVID-19 disease.

Biological information for lanadelumab

Lanadelumab (Takhyzro®, formerly DX-2930 or SHP643) is a recombinant, Chinese hamster ovary (CHO) cell expressed, fully human immunoglobulin G subclass 1 (IgG1), kappa light chain, monoclonal antibody. Lanadelumab is a potent and specific inhibitor (inhibition constant [Ki]=0.12 nM) of the proteolytic activity of active plasma kallikrein (PKa) (Kenniston et al., 2014). Lanadelumab has been approved by the FDA and EMA for the prophylactic treatment in patients with type I or II hereditary angioedema (HAE), a rare and life-threatening disease (Longhurst and Cicardi, 2012). The activity, potency, and specificity of lanadelumab have been demonstrated in vitro and ex vivo. Preclinical, in vitro pharmacology potency studies indicated that PKa inhibition in rat and cynomolgus monkey (Ki of 170 pM and 69 pM, respectively) was similar to human PKa inhibition (Ki of 125 pM) (Busse et al., 2018; Kenniston et al., 2014). Lanadelumab possesses high specificity for PKa and does not inhibit 20 other serine proteases tested at concentrations of either 1 or 5 µM, including activated factor XI, which has significant homology to PKa. In an in vivo model of edema, lanadelumab inhibited the induction of paw edema in rats in a dose-dependent manner.(Kenniston et al., 2014) Lanadelumab does not have adverse effects on vital functions of the central nervous system (CNS), cardiovascular (CV) system, or respiratory system based on safety pharmacology endpoints incorporated in the pivotal repeat-dose studies in rats and cynomolgus monkeys (https://www.ema.europa.eu/en/documents/assessment-report/takhzyro-epar-public-assessment-report_en.pdf).

In non-clinical studies, the pharmacokinetics of lanadelumab exhibited low clearance, low volume of distribution, and long half-lives (rats approximately 3 days on average and cynomolgus monkeys approximately 10 days on average), with an approximate subcutaneous (SC) bioavailability of 66% in cynomolgus monkeys.(Kenniston et al., 2014) Maximum concentrations following SC administration were reached 1 to 4 days and 2 to 4 days post dose in rats and cynomolgus monkeys, respectively. The nonclinical toxicity studies demonstrated that SC administration of lanadelumab to rats (28 days) and cynomolgus monkey (up to 6 months) was well tolerated with no evidence of organ toxicity at weekly doses of up to and including 50 mg/kg, the highest dose administered and the no-observed-adverse-effect-level (NOAEL) in all nonclinical studies. At the NOAEL in the 6-month cynomolgus

monkey study, exposure margins based on maximum concentration occurring at t_{max} (C_{max}) and area under the drug concentration-time curve (AUC) were approximately 21- and 23-fold higher, respectively, than those observed at the clinical dosage of 300 mg every 2 weeks (q2wks; DX-2930-03) (https://www.ema.europa.eu/en/documents/assessment-report/takhzyro-epar-public-assessment-report_en.pdf).

The clinical development program for lanadelumab consists of one pivotal study supported by interim data from a long-term open label extension study and data from a "proof-of concept"/dose-finding study (Banerji et al., 2017, 2018; Riedl et al., 2017). Choice of lanadelumab dosing for the pivotal phase 3 (HELP) study was accomplished by the sponsor using the safety, pharmacokinetic and pharmacodynamic data generated in the phase 1 studies DX-2930-01 (healthy subjects) and DX-2930-02 (subjects with HAE). The planned range of lanadelumab dosing for the clinical development program was based upon estimation of the level of plasma kallikrein (pKal) inhibition necessary to attain effective prophylaxis against HAE attacks. It was hypothesized that the necessary molar concentration of lanadelumab would correspond to the average Cmax attained following administration of ecallantide, a biologic pKal inhibitor. The Cmax obtained following ecallantide administration is approximately 80 nM or 586 ng/mL.

The pivotal study, DX-2930-03, was a phase 3, multicenter, randomized, double-blind, placebo-controlled HAE prevention study with a 26-week long treatment duration. One hundred and twenty-five subjects (115 adults and 10 adolescents aged 12-≤18) were included in study DX-2930-03 and treated with placebo (N=41) or lanadelumab (N=84). Among the adolescents, 4 were treated with placebo and 6 received lanadelumab.(Banerji et al., 2018) The main eligibility criteria were subjects ≥12 years of age with HAE and at least 1 investigator confirmed HAE attack per 4 weeks. The number of investigator- confirmed HAE attacks during the treatment period was reduced in all lanadelumab treatment arms compared to placebo (p<0.001 for all lanadelumab arms vs placebo). The relative risk reduction compared to placebo ranged from 73% in the 300 mg every 4 weeks arm to 87% in the 300 mg every 2 weeks arm, corresponding to a decrease of approximately 1.5 attack/4 weeks in the 150 mg q4wks and 300 mg every 4 weeks arms and 1.75 attacks/4 weeks in the 300 mg every 2 weeks treatment arm based on LS mean HAE attack rate in the four treatment arms.

In summary of the above:
We believe lanaludumab is the drug of choice over icatibant to deployed in prevention of ARDS in patients with COVID-19 disease. We have chosen the intravenous route of administration over the licensed subcuteanous route for direct clinical effect. We believe this approach is safe.

Timing of drug administration (target population)

The main goal of treating patients with moderate COVID-19 disease in the hospital is to prevent the progression to severe disease that frequently progresses to ARDS and requires prolonged mechanical ventilation. We plan to include patients with moderate or severe disease for treatment with lanadelumab according to the SWAB definition for COVID-19 disease. Treating patients who are hospitalized with respiratory symptoms as early as possible with a dosing regimen above will optimize our ability to test modulation of the kallikrein/kinin pathway as potential treatment strategy in patients with COVID-19.

1.3 Primary objectives

- To deliver the proof-of-concept of relieve of symptoms by using lanadelumab in patients with moderate to severe COVID-19 disease
- To demonstrate the safety of lanadelumab given as intravenous administration in patients with COVID-19 disease.

2. Study design

The proposed study is an open-label, randomized, intervention study in patients tested positive for SARS-CoV2 on the ward. Fourty patients will be enrolled in a 1:1 randomized fashion for intervention (lanadelumab administration) versus controls (standard treatment). To be able to randomize fourty patients within the study duration, a multi-center approach is chosen.
We have chosen to perform this trial in patients that are Sars-COV2 positive as we believe they can directly benefit from this intervention. As such it merits preference over testing this drug first in healthy volunteers to demonstrate the safety of intravenous administration and then move into patients with COVID19 disease. We argue this is best done in a combined fashion.

3. Subject population

3.1 Number of subjects and subject selection

Fourthy patients will be randomized 1:1 for the intervention with lanadelumab iv. Twenty SARS-COV2 positive patients treated with lanadelumab 300 mg IV on day 1, followed by a second dose of lanadelamub 300 mg IV on day 4. The other 20 lanadelumab patients will receive the same treatment algorithm while NOT receiving lanadelumab. The estimated number of patients being treated for COVID disease is difficult to predict. Currently about 20% of the COVID-19 patients are hospitalized and out of these patients about 20% is admitted to the ICU.

These will be patients from the following centers:

- Amsterdam UMC, Amsterdam
- Radboud University Medical center, Nijmegen,
- University Medical Center Utrecht, Utrecht,
- Rijnstate Hospital, Arnhem
- Flevoziekenhuis, Almere

3.2 Inclusion criteria

Subjects must meet all of the following inclusion criteria to be eligible for participation in this study:

1. Patient is SARS-COV2 positive
2. Without oxygen a saturation below 90% or / and At least 3L/min oxygen dependent
3. Patient is 16 years and older

3.3 Exclusion criteria

Subjects who meet any of the following criteria will be excluded from participation in this study:

1. Has previously participated in this study
2. Acute myocardial or cerebral ischemic event at time of enrolment
3. Receiving an agent that is specified as an intervention in this domain as a usual medication prior to this hospitalization will exclude a patient from receiving that agent
4. A baseline alanine aminotransferase or an aspartate aminotransferase that is more than five times the upper limit of normal
5. Patient is known hypersensitive to full human monoclonal antibodies.
6. Patient is pregnant or breast feeding

3.4 Study duration

The expected duration of the study for an individual patient will be as long as the patient is hospitalized. The estimated length of time needed to complete the entire study will be one month.

3.5 Removal of subjects from the study

The investigator may terminate participation if:

- There is violation of protocol
- It is in the best interest of the subject
- Patients that are transferred to the ICU will be followed up for safety and if possible samples will be taken, but are considered to have reached a study endpoint.

Subjects dropping out before the final dose has been given, will not be replaced. Patients are regarded evaluable after both treatment days are completed.

3.6 Replacement of subjects

Subjects will be replaced if an incorrect lanadelumab dose was given, other dropouts will not be replaced in case of dropouts as the study has an explorative nature.

3.7 Stopping rules for the study

The study will be stopped if at least 1 patient experiences a severe adverse event that is attributable to lanadelumab treatment and evaluation will take place before the next subject is treated or whether the second dose of lanadelumab can be given. A semi-sequential design will be deployed. This means that we will start with a single individual to be entered into the study. A second subject can enter the study 24 hours after the first subject has started. After the second participant has received the first dose without severe complications, the 3rd to 5th participant may be recruited.

4. Treatment

In the current study subjects will be treated with lanadelumab 300 mg IV on day 1 followed by a second dose of lanadelumab 300mg IV on day 4.

4.1 Dose rationale and rate of infusion

We refer to the attachment with the results from the modeling and simulation data. This provides the necessary rationale to administer 300 mg IV followed by a second dose of 300 mg IV on day 4 of treatment.

Lanadelumab will be administered in a similar way as all current monoclonal antibodies are given. That is a ramp up scheme in which the infusion rate is steadily increased. For lanadelumab we will follow the following scheme. In the end the total volume will be administered over a period of 1 hour.

Dose	duration	Rate
First 30 mg = 10 mL	20 minutes	30 mL/hour = 90 mg/hour
30-100 mg (70 mg) = 23.33 mL	20 minutes	70 mL/hour = 210 mg/hour
100-300 mg (200 mg) = 66.67 mL	20 minutes	200 mL/hour = 600 mg/hour

300 mg is diluted in 100 mL NaCl 0.9% to a concentration of 3 mg/mL

4.2 Drug interactions

Drug interactions are not expected for monoclonal antibodies as is confirmed by the details in the EPAR of lanadelumab.

4.3 Safety and potential risks

For general safety information we refer to the SmPC of lanadelumab.
Briefly stated it has the following information:
Hypersensitivity is an important identified risk and is discussed in the warnings and precautions of the reference labelling. In case of a severe hypersensitivity reaction, administration of TAKHZYRO must be stopped immediately and appropriate treatment must be initiated.

Immunogenicity is an important potential risk for lanadelumab and is monitored closely. The known potential risks of disordered coagulation associated with monoclonal antibodies have been characterized and no unexpected signals were identified.

The most commonly occurring adverse events with greater frequency in the lanadelumab treatment groups were injection site reactions (34.1% placebo, 52.4% lanadelumab) and dizziness (0% placebo, 6.0% lanadelumab). Less common reactions in the lanadelumab group compared to placebo include hypersensitivity (1% vs. 0%), increased liver enzymes (2% compared to 0%). An open-label extension study to evaluate the long-term safety and efficacy of lanadelumab for prevention of HAE attacks is ongoing. (Riedl et al., 2017). Pharmacokinetic analyses showed that the use of concomitant medications such as analgesic, antibacterial, antihistamine, anti-inflammatory, and antirheumatic medications used in the treatment of HAE had no effect on the pharmacokinetic properties of lanadelumab, suggesting that the drug-drug interaction potential of those evaluated drug classes is low. Similar observations were made for rescue medications, including plasma-derived and recombinant C1-INH, ecallantide, and icatibant, used for acute treatment of HAE attacks.

In this study two dosages of 300 mg intravenous lanadelumab are given on day 1 and day 4. The cumulative dose of 600 mg per week is higher than the current licensed dose for prevention of HAE. Even with a second dose given on day 4 of treatment the predicted drug concentrations remain far below the maximal tolerable dose. [see pop-PKPD modeling file with simulations]. We do not expect additional toxicity of the approach chosen.

Additional safety information to support for lanadelumab given as intravenous administration
Lanadelumab is currently marketed under TAKHZYRO tradename as a sterile, preservative-free, clear to slightly opalescent, colorless to slightly yellow solution currently approved in a single-dose glass vial for subcutaneous (SC) injection only; Injection: 300 mg/2 mL (150 mg/mL) solution. The Sponsor intends to use currently approved TAKHZYRO vials to be diluted in 100 ml saline solution bags for IV administration (Luo et al, 2020). Information on biostability after dilution is provided in the document "DX2930 Matl Compatibility and Cond of Use Stability Summary IV Tox MD 04AUG2014".

Based on the mechanism of action, PK/PD extrapolations, prior experience with lanadelumab in clinical trials, and supported safety profile based on the existing clinical package, the recommended dose is one infusion of lanadelumab 300 mg IV and a second dose of lanadelumab 300mg IV on day 4 (see pop-PKPD modeling and simulation file).

Preclinical safety information data after intravenous administration: (DATA ON FILE OF TAKEDA)

In addition to toxicology studies conducted via subcutaneous administration, lanadelumab was also evaluated in a single-dose IV infusion dose-range finding study with 4 weeks of recovery and a 4-week IV infusion study with 4 weeks of recovery in cynomolgus monkeys.

The objective of the non-GLP single-dose study was to evaluate the tolerability and TK profile of lanadelumab when administered once by IV infusion (approximately 10 minutes) to male and female cynomolgus monkey, as well as to evaluate the recovery, persistence, or progression of any effects following a minimum of a 28-day observation period. Lanadelumab (in a vehicle consisting of 30 mM sodium phosphate, 8.6 mM citric acid, 50 mM histidine, 90 mM sodium chloride, pH 6.0, and 0.01% polysorbate 80) was administered as a single IV infusion to cynomolgus monkeys (n=2 per sex per group) at doses of 5, 25, or 50 mg/kg, at a dose volume of 5 mL/kg. Parameters evaluated included mortality and moribundity, clinical observations, detailed physical examinations, body weight, hematology, coagulation, serum chemistry, and urinalysis. Toxicokinetic evaluations were also conducted. All animals survived to the end of the study. There were no lanadelumab-related clinical observations or effects on body weights. Hematology, coagulation, serum chemistry, and urine parameters were unaffected by lanadelumab administration.

Based on the results of this study, a single IV infusion of lanadelumab at dose levels of 5, 25, and 50 mg/kg to cynomolgus monkeys was well tolerated at all dose levels evaluated. A dose of 50 mg/kg corresponded to mean AUClast values of 132000 and 143000 µg·hr/mL and Cmax values of 1300 and 1010 µg/mL for males and females, respectively. In our proposal we will administer a dose of around 5 mg/kg (300 mg).

The objective of a GLP study repeat-dose study was to evaluate the toxicity potential and TK profile of lanadelumab when administered once weekly for 4 weeks by IV infusion (approximately 10 minutes) to male and female cynomolgus monkeys, as well as to evaluate the recovery, persistence, or progression of any effects following a minimum of a 28-day recovery period.

In this study, vehicle (30 mM sodium phosphate, 8.6 mM citric acid, 50 mM histidine, 90 mM sodium chloride, pH 6.0, and 0.01% polysorbate 80) or lanadelumab at doses of 5, 25, or 50 mg/kg was administered via IV infusion once weekly for 4 weeks (Study Days 0, 7, 14, 21, and 28 [5 total doses]) to groups of 5 (Groups 1 and 4) or 3 (Groups 2 and 3) cynomolgus monkeys per sex. Following the final

dose administration, 3 animals per sex per group were euthanized; the remaining 2 animals per sex from Groups 1 and 4 were euthanized following a 28-day recovery period. The dose volume for all groups was 5 mL/kg. Parameters evaluated included mortality and moribundity, clinical examinations, detailed physical examinations, body weight, ophthalmology, ECG, heart rate, blood pressure and respiration rate, hematology, coagulation, serum chemistry, urinalysis, organ weights, and macroscopic and microscopic histological examinations. Toxicokinetic and ADA evaluations were conducted in all animals.

All animals survived to the scheduled necropsies. There were no lanadelumab-related clinical observations or effects on body weights, clinical pathology parameters, ECG parameters, blood pressure, respiration rates, or organ weights. There were no lanadelumab-related ophthalmic, gross, or microscopic findings.

A total of 72 samples collected on Study Days 0, 29, and 57 were screened for the presence of anti-lanadelumab antibodies in cynomolgus monkey sodium citrate plasma. Of these, 7 presumptive positive samples were subjected to confirmatory analysis; 2 of those samples (50 mg/kg male at Study Day 57; and 50 mg/kg female at Study Day 0) were confirmed positive for the presence of anti-lanadelumab antibodies. The control group female did not confirm positive at the subsequent collection (Study Day 29). Presence of ADAs had no impact on exposure in cynomolgus monkey plasma.

Based on the results of this study, once weekly IV infusion (approximately 10 minutes) of lanadelumab at doses of 5, 25, or 50 mg/kg for 4 weeks (Study Days 0, 7, 14, 21, and 28 [5 total doses]) was well tolerated in cynomolgus monkey. There were no adverse lanadelumab-related findings noted at any dose level and the highest dose of 50 mg/kg was determined to be the NOAEL. This dose corresponded to mean Study Day 21 AUClast values of 127000 or 119000 µg·hr/mL and Cmax values of 1650 or 1630 µg/mL, for males and females respectively.

The nonclinical development program for lanadelumab as described in this section and listed in Appendix 2 demonstrate that lanadelumab did not have adverse effects on vital functions or produce adverse target organ pathologies in rats or cynomolgus monkeys at all tested doses.

<u>Safety information of drugs targeting the ACE2 domain:</u>
For patients with COVID-19 disease we can indicate the following with regards to safety in the ACE2 domain: Icatibant treatment was well-tolerated in all 10 patients who received icatibant for COVID-19 disease. The adverse event most commonly reported was pain at the site of injection. These effects were temporarily and resolved without need of treatment. A slight increase of less than threefold upper limit of normal of AST and ALT was observed with icatibant treatment. This side effect is commonly observed in patients treated with icatibant. All liver function test resolved spontaneously and no additional intervention was needed. No serious adverse events were observed.
We do not expect specific COVID driven toxicity.

5. Trial drugs

5.1 Identity of investigational product(s)

Lanadelumab has a marketing authorisation in the European Union. Takeda will provide sufficient quantities of fully characterized investigational medicinal products prepared in accordance with Good Manufacturing Practice (GMP), properly packaged and labelled.

The trial drugs will be accounted for in a drug inventory and stored at appropriate conditions (intravenous solution has to be stored in the refrigerator preferably in a locked safe, until used in the trial. Excess trial medication will be returned to the sponsor. The study pharmacist will supervise the storage of all trial medication.

The following formulations will be used:

Name : Takhzyro®

Active ingredient : lanadelumab

Excipients : Disodium phosphate dihydrat, Citric acid monohydrate, Histidine, Sodium chloride, Polysorbate 80, Water for injections

Dosage form : 2 ml of solution in a vial (type I glass) with a coated butyl rubber stopper and an aluminium seal with violet flip-off cap. This will be reconstituted with sodiumchlorde 0,9% to a final concentration of 3.0 mg/mL. Lanadelumab will be given over an inline filter of 0.2 mu.

Strength : 300mg

Manufacturer : Takeda / Shire.

5.2 Packaging and labelling of trial medication

Trial medication will be supplied as the commercially available lanadelumab. These vials will be used during the trial. Since this is an open trial, blinding procedures are not applicable. The containers will be labelled with: sponsor’s name and contact details / phone number, protocol number, name study, drug name, number of capsules and strength dose, instructions for use, batch number, storage conditions, expiry date, “For clinical trial use only”, “Keep out of reach of children” in concordance with GMP annex 13 legislation. In addition, the investigational staff will label the containers and package with subject code / randomisation number.

5.3 Method of assigning subjects to treatment groups

Subject will be randomized in a 1:1 ratio to receive lanadelumab or standard clinical management without drug intervention. Each group will contain 20 participants.

Randomization will be done by Castor. Each patient will be entered into the Castor system and randomization will be allocated immediately. This is a standard procedure as deployed in other trials.

Each site has drug on site for at least one patient and therefor randomisation to the intervention arm can be acted upon immediately. Considering the small sample size there will be no stratification.

5.4 Selection and timing of dosing and dietary

Explanation of the dosing scheme is provided in the protocol. No restrictions are applicable to dietary.

5.5 Treatment Compliance

All administration of medication is supervised and recorded by the study personnel. The Investigator or his/her designee will maintain an adequate record of the receipt and distribution of all study supplies. These forms will be available for inspection at any time.

5.6 Drug accountability

The investigator or designee (i.e., pharmacist) is responsible for ensuring adequate accountability of all used and unused study drugs. Dispensing records will document quantities received from the CTU and quantities dispensed to subjects, including lot number, date dispensed, subject identifier number, subject initials, and the initials of the person dispensing the medication.

At study initiation, the monitor will evaluate and approve the site's procedure for study drug disposal/destruction in order to ensure that it complies with GCP requirements. If the site cannot meet GCP requirements for disposal/destruction, arrangements will be made between the site and the sponsor or its representative, for return of unused study drug supplies.

During the study the drugs will be stored at the site. Drug storage will also be evaluated at the initiation of the study.

Prior to disposal/destruction or return of used, unused, and depleted containers, final drug accountability and reconciliation will be performed by the monitor.

All drug supplies and associated documentation will be regularly reviewed and verified by the monitor.

5.7 Prior and Concomitant Therapy

For two weeks preceding day 1 and during the study, all concomitant medication will be recorded in the subject's Case Report Form (CRF). All medication is allowed during this trial except for experimental drugs listed in the exclusion criteria.

6. Study procedures

6.1 Inclusion Screening and screening during treatment

At inclusion screening the medical history of the subject will be verified and documented to establish eligibility for inclusion. In table 2 a summary is listed of the parameters that should be collected at baseline and while on treatment.

Table 1: summary of study procedures during the study

	At inclusion	Day 1	2	3	4	5	6	7	8-14	>14	
Demographics[a]	X	X									
Physical examination[b]	X	X									
Laboratory tests[c]	X	X	X	X	X	X	X	X	X	X	X
Administration lanadelumab intervention group only		X			X						
Oxygen need[d]	X	X	X	X	X	X	X	X	X	X	X
Concomitant medication	X	X	X	X	X	X	X	X	X	X	X
Clinical outcome	X	X	X	X	X	X	X	X	X	X	X
PK[e]		X	X	X	X	X	X	X	X		
PD & in depth sampling[f]		X			X						

a: sex, age, BMI, days of illness, Charlson Comorbidity Index (CCI), comedication, CT severity score, oxygen saturation without supplementation, oxygen supplementation need, respiratory rate at baseline.
b: respiratory rate, oxygen saturation, and supplemental oxygen.
c: hemocytometry, WBC differentiation, D-dimer, ferritin, CRP, and albumin. EDTA plasma 10 mL and citrate 4 mL for plasma kallikrein metabolites.
d: change in oxygen need and oxygenation expressed as absolute number of liters per hour using a target oxygenation of above 94% as a marker of pulmonary status. ICU admission after admission to the ward, changes in D-dimer relative to baseline, PKa metabolites changes relative to baseline, safety.
e determination of lanadelumab plasma concentration (pk outcome, intervention group only) and bradykinin markers (PD outcome.) PK after first lanadelumab adminstration: T1=12-24; T2 = T1 + 24 (+/-6 hours); T3= T1+ 48 (+/-6 hours); T4= T1 + 72 (+/-6 hours); T5= T1 + 96(+/-6 hours); T6 = T1 + 120 (+/-6 hours); T7 = T1 + 144 (+/-6 hours) and then daily. If only one lanadelumab dose is given PK will be sampled up to and including day 7, or until patient is dismissed from the hospital whichever comes first.
f Determination of bradykininemarkers and in-depth sampling. Labsampling day 1 needs to be done before first administration of lanadelumab. Day 4 has a window of +/- 2 days if day 4 is in the weekend. Blisters for day 1 and 4 include 2x 10 ml EDTA, 2x 4 ml citrate and 1 PAXgene for in depth labanalysis., See also the labmanual.

Oxygen suuply treatment procedures:

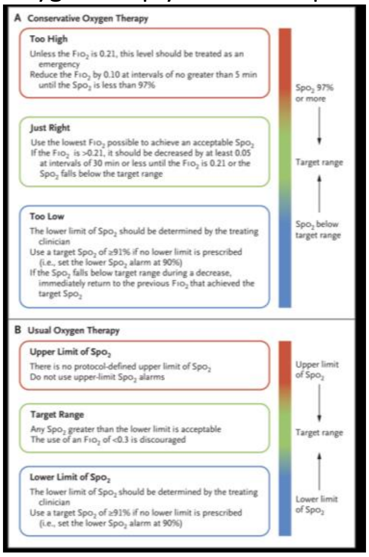

A well-established standardized protocol published in NEJM which follows the approach in the flow diagram of conservative oxygen therapy, which is based on titrating on the lowest acceptable SpO2 with 30 min intervals will be used. (The ICU-ROX Investigators and the Australian and New Zealand Intensive Care Society Clinical Trials Group. N Engl J Med 2020;382:989-998)

Data and outcome variables:

Data on clinical parameters including respiratory rate, oxygen saturation, and supplemental oxygen are registered before and after administration of lanadelumab and then daily. Changes in oxygen support requirement and clinical symptoms are recorded. Laboratory tests are performed during the study: hemocytometry, WBC differentiation, D-dimer, ferritin, CRP, and albumin (Table 1).

Kinins, biomarkers, and functional immunological profiling will be assessed in the fourty randomized patients with EDTA plasma (10ml), PBMCs (20ml EDTA), mass spectrometry (2ml blood in specialized medium tube), and citrate plasma (8ml), with a total volume of 40ml per in depth sampling.

6.2 Safety assessments

Safety assessments will be recorded as part of this trial.

6.3 Safety reporting

The methods of this study are considered as limited risks. In the unlikely event that a patient dies due to the lanadelumab treatment this will be reported to the ethical committee via toetsingonline.nl. The investigator must assess the causality of death. In case there is clear evidence that suggests a causal relationship this will be reported to the sponsor on a SAE form.

1.1 Temporary halt for reasons of subject safety

In accordance to section 10, subsection 4, of the WMO, the sponsor will suspend the study if there is sufficient ground that continuation of the study will jeopardise subject health or safety. The sponsor will notify the accredited METC without undue delay of a temporary halt including the reason for such an action. The study will be suspended pending a further positive decision by the accredited METC. The investigator will take care that all subjects are kept informed.

Adverse events (AEs)

Adverse events are defined as any undesirable experience occurring to a subject during the study, whether or not considered related to the investigational product. All adverse events reported spontaneously by the subject or observed by the investigator or his staff will be recorded, meeting all of the following criteria:

- **Severity: Grade 2 or higher, or grade 1 lasting longer than 1 week, graded by the CTCAE (Common Terminology Criteria for Adverse Events, v5.0).**
- **Causality: there needs to be a reasonable suspicion of the AE being an effct of the study medication.**

Serious Adverse events (SAEs)

A serious adverse event is any untoward medical occurrence or effect that

- results in death;
- is life threatening (at the time of the event);
- requires hospitalisation or prolongation of existing inpatients' hospitalisation, admittance to the Intensive Care Unit due to respiratory insufficiency will not be reported as an SAE;
- results in persistent or significant disability or incapacity;
- is a congenital anomaly or birth defect; or
- any other important medical event that did not result in any of the outcomes listed above due to medical or surgical intervention but could have been based upon appropriate judgement by the investigator.

The investigator in a participating center will report all SAEs to the coordinating investigator without undue delay after obtaining knowledge of the events.

The sponsor will report the SAEs through the web portal *ToetsingOnline* to the accredited METC that approved the protocol, within 7 days of first knowledge for SAEs that result in death or are life threatening followed by a period of maximum of 8 days to complete the initial preliminary report. All other SAEs will be reported within a period of maximum 15 days after the sponsor has first knowledge of the serious adverse events.

Suspected unexpected serious adverse reactions (SUSARs)

Adverse reactions are all untoward and unintended responses to an investigational product related to any dose administered.

Unexpected adverse reactions are SUSARs if the following three conditions are met:

1. the event must be serious;
2. there must be a certain degree of probability that the event is a harmful and an undesirable reaction to the medicinal product under investigation, regardless of the administered dose;
3. the adverse reaction must be unexpected, that is to say, the nature and severity of the adverse reaction are not in agreement with the product information as recorded in:
   - Summary of Product Characteristics (SPC) for an authorised medicinal product;
   - Investigator's Brochure for an unauthorised medicinal product.

The investigator in a participatingcentre will report the SUSAR to the coordinating investigator.
The coordinating investigator will report expedited the following SUSARs through the web portal *ToetsingOnline* to the METC:

- SUSARs that have arisen in the clinical trial that was assessed by the METC;
- SUSARs that have arisen in other clinical trials of the same sponsor and with the same medicinal product, and that could have consequences for the safety of the subjects involved in the clinical trial that was assessed by the METC.

The remaining SUSARs are recorded in an overview list (line-listing) that will be submitted once every half year to the METC. This line-listing provides an overview of all SUSARs from the study medicine, accompanied by a brief report highlighting the main points of concern.

The expedited reporting of SUSARs through the web portal Eudravigilance or ToetsingOnline is sufficient as notification to the competent authority.

The sponsor will report expedited all SUSARs to the competent authorities in other Member States, according to the requirements of the Member States.

The expedited reporting will occur not later than 15 days after the sponsor has first knowledge of the adverse reactions. For fatal or life threatening cases the term will be maximal 7 days for a preliminary report with another 8 days for completion of the report.

For individual sites, the local investigator completes the SAE report in Castor and providing as much detailed information as known and relevant to the event within 24 hours of discovery of the event.
The coordinating investigator will automatically be notified via Castor by email when a new SAE is reported in Castor. If there are technical issues with Castor the local investigator complets the SAE report on paper. A template of this report will be provided in de ISF. The sponsor will report all SAEs using the CCMO module 'ToetsingOnline' to the accredited METC that approved the protocol. By means of this website notifications will be sent to the relevant authorities (METC/LAREB/EudraVigilance). The reporting will occur within 15 days after the investigator has first received information on the SAE. For fatal or life-threatening cases a preliminary report will be offered within 7 days followed by a complete report within 8 days. SUSARs will be electronically reported via ToetsingOnline and the trial coordinator will communicate all SUSARs to the independent monitor of this study.

7. Data analysis

7.1 Sampling design and sample size justification

This is pragmatic study to deliver a proof of concept on the use of lanadelumab in COVID patients on the ward. No formal sample size has been performed.

7.2 Data collection and data management

All data obtained in the study described in this protocol will be anonymously recorded on CRFs by health professionals who are involved with the treatment of the subjects.

7.3 Statistical analysis

Statistical analysis will be performed using descriptive statistics for tabulation of population as well as results. Prof. Kit Roes from our department of health evidence will help with the statistical analysis. Appropriate test will be deployed.

For the primary endpoint we intend to collect repeated measurements of oxygen use in L/min and therefore any significant decline in a patient's health should be reflected in these repeated measurements. We acknowledge that missing data due to death is a potential source of bias, particularly if oxygen is stopped at the point where a significant decline is observed. Therefore, a worst-case scenario sensitivity analysis, in which the highest value of oxygen recorded for any patient is imputed for patients with missing data following their death. Other valid methods to account for missing data due to death will be investigated and included in the Statistical Analysis Plan.

8. Structured Risk Analysis

This study uses lanadelumab, which is an agent licensed for prophylaxis of hereditary angioedema. The dosages and clinical indications used in this trial are similar to the licensed dosages; therefore no potential harmful risks are expected in this cohort.

Lanadelumab will be given intravenous contrary to the licensed subcutaneous administration. This route of administration has been demonstrated safe at higher dosages then we will intend to use in primates.

The frequency of two dosages within a time frame is supported by population PK and PD modelling.

The burden of the patient is identical to studies previously performed in the same cohort (icatibant, reviewed by the ethics committee). We strongly believe the burden for the patient as well as the risk for severe adverse events is reduced to an absolute minimum.

We have chosen to perform this trial in patients that are Sars-COV2 positive as we believe they can directly benefit from this intervention. We consider the risk of this trial to be minimal. A similar approach to target the bradykinin pathway in 10 patients with COVID disease did not reveal specific serious adverse events. Furthermore, lanadelumab has been demonstrated safe in both healthy volunteers and patients with HAE at dosages that exceed the dosages proposed in this trial. The dosages used are strongly supported by modeling and simulation.

A possible risk could be introduced by administration of lanadelumab via intravenous infusion. As there are no harmful excipients used in lanadelumab formulation, and the drug is a full human monoclonal antibody, we expect the risk to be minimal as well with regards to this aspect.

The risk-classification is assessed as low to the patient population receiving study drug at the current regimens. Safety data on the use of higher dose are published and very-well defined. There is no attributable risk for the application of the study protocol to the COVID patients.

9. ETHICAL CONSIDERATIONS / RESPONSIBILITIES

9.1 Investigator Responsibilities

9.1.1 Good Clinical Practice

The investigator will ensure that this study is conducted in full compliance with the most recent version of principles of the "Declaration of Helsinki", ICH guidelines, or with the laws and regulations of the country in which the research is conducted, whichever affords the greater protection to the study subject.

9.1.2 Institutional Review Board (IRB)/Independent Ethics Committee (IEC) Approval

This protocol and any accompanying material to be provided to the subject (such as advertisements, subject information sheets, or descriptions of the study used to obtain informed consent) will be submitted, by the investigator, to an IEC. Approval from the committee must be obtained before starting the study and should be documented in a letter to the investigator specifying the protocol number, protocol version, documents reviewed, and date on which the committee met and granted the approval.

Any modifications made to the protocol after receipt of IEC approval must also be submitted to the committee for approval prior to implementation.

9.1.3 Informed Consent

It is the responsibility of the investigator to obtain written informed consent from each individual participating in this study after adequate explanation of the aims, methods, objectives, and potential hazards of the study and prior to undertaking any study-related procedures. The investigator must utilize an IEC-approved consent form for documenting written informed consent. Each informed consent will be appropriately signed and dated by the subject and the persons obtaining consent.

If, due to infection risk, obtaining written informed consent is not possible, the patient will be asked to consent verbally, with an independent witness present. This procedure and the name of the witness will be registered in the electronic patient file and identification log. At the moment it is feasible to obtain written informed consent (i.e. when the infection risk is reduced), this has to be obtained as well. A remark will be made on the written informed consent that verbal informed consent also has been given with the date of verbal informed consent.

For the historical controls asking the patient for permission in advance is not possible. This is because there is a disproportionate relationship between the time investment involved in requesting permission and the time involved in conducting the study.

9.1.4 Benefits and risks assessment, group relatedness

For a number of reasons administration of the intravenous lanadeluamb can lead to problems with administration. Pharmacokinetic properties and information about safety have only been derived from modelling and simulation.

There are currently no treatment options for patients with COVID19. As lanadelumab subcutanous is expected to result in a substantial delay in effect due to the slow absorption, intravenous administration is important. Therefore, we want to investigate this alternative safe method to administer lanadelumab. The potential value of this research is to provide both the proof of concept as well as safety of treatment in COVID disease..

This study will be performed in patients with COVID disease as recommended by the guidelines for bioequivalence. Lanadelumab is well tolerated in other patient cohorts. There is a mild side effect profile as also

demonstrated in large phase III trials. Moreover, to limit the risk of side effects, this study is designed as two dose study.

The study participants are subjects ≥ 18 years. The safety and efficacy of intravenous lanadelumab in children and adolescents aged less than 18 years have not yet been established.

For pharmacokinetic purposes and safety assessment, blood samples will be collected. The total blood volume taken will be approximately 100-150 mL. During the days that blood samples will be collected for biomarker and PK-information, an intravenous cannula will be inserted to facilitate blood sampling and limit the amount of venous punctures.

9.1.5 Incentives

Subjects will receive no fee for this trial.

9.1.6 Confidentiality

The investigator must assure that subjects' anonymity will be strictly maintained and that their identities are protected from unauthorized parties. Only an identification code (i.e., not names) should be recorded on any form submitted to the sponsor and IEC. The investigator must keep a screening log showing codes, for all subjects screened and for all subjects enrolled in the study.

This information is not to be disclosed to any third party (except employees or agents directly involved in the conduct of the study or as required by law) without prior written consent from the Investigator. The investigator further agrees to take all reasonable precautions to prevent the disclosure by any employee or agent of the study site to any third party or otherwise into the public domain.

9.1.7 Study Files and Retention of Records and Samples

The investigator must maintain adequate and accurate records to enable the conduct of the study to be fully documented and the study data to be subsequently verified. These documents should be classified into 2 separate categories: (1) investigator's study file and (2) subject clinical source documents. Castor EDC will be used as datamanagement system.

The investigator's study file will contain the protocol/amendments, CRF and query forms, IEC and governmental approval with correspondence, informed consent, drug records, staff curriculum vitae and authorization forms, and other appropriate documents and correspondence.

Subject clinical source documents (usually defined by the project in advance to record key efficacy/safety parameters independent of the CRFs) would for example include subject hospital/clinic records, physician's and nurse's notes, appointment book, original laboratory reports, electrocardiogram (ECG), X-ray, pathology and special assessment reports, consultant letters, screening and enrolment log, etc.

All clinical study documents must be retained by the investigator for at least 5 years. The investigator must be notified prior to destroying any clinical study records.

After agreement of the subject, samples will be stored until 5 years after the start of this study in the laboratory of the pharmacy department of the Radboud university medical centre. The principle investigator is responsible for the storage. This material will be stored to study the pharmacokinetics or pharmacological questions of lanadelumab in the future. After this period the material will be destroyed. Whenever there is an intention to use samples during the period of 5 years for research that does not directly relate to the current research or in the case that coincidental findings can occur, the ethical commission will be consulted for permission.

9.1.8 Case Report Forms

For each subject enrolled, a CRF must be completed and approved by the principal investigator or co-/sub-investigator within a reasonable time period after data collection. This also applies to records for those subjects who fail to complete the study (even during a screening period if a CRF was initiated). If a subject withdraws from the study, the reason must be noted on the CRF. If a subject is withdrawn from the study because of a treatment-limiting adverse event, thorough efforts should be made to clearly document the outcome.

9.1.9 Inspections

The investigator should understand that source documents for this study should be made available to regulatory authority or health authority inspectors.

9.1.10 Protocol Compliance

The investigator is responsible for ensuring the study is conducted in accordance with the procedures and evaluations described in this protocol.

9.1.11 Insurance Cover

Since Radboudumc is the sponsor of this study, insurances will be arranged by the Radboudumc.

According to the Dutch law (WMO) the sponsor is obliged to have an insurance for compensation of subjects entered in clinical studies in the Netherlands who experienced study related injury or death. Therefore, Radboudumc, The Netherlands, has arranged a liability insurance, which is in accordance with article 7, subsection 6 of the WMO.

The Radboudumc also has an insurance providing cover for damage to research subjects through injury or death caused by the study (in accordance with the legal requirements in The Netherlands: Article 7 WMO and the Measure regarding Compulsory Insurance for Clinical Research in Humans).

The insurance is described in detail in the written subject information.

9.2 Sponsor Responsibilities

9.2.1 Protocol Modifications

Protocol modifications, except those intended to reduce immediate risk to study subjects, may be made only by the sponsor. All protocol modifications must be submitted to the IEC in accordance with local requirements. Approval must be obtained before changes can be implemented.

9.2.2 Study Progress report

The sponsor/investigator will submit a summary of the progress of the study to the accredited METC once a year. Information will be provided on the date of inclusion of the first subject, numbers of subjects included and numbers of subjects that have completed the study, serious adverse events/ serious adverse reactions, other problems, and amendments.

9.2.3 Study Report and Publications

The sponsor will notify the accredited METC and the competent authority of the end of the study within a period of 90 days. The end of the study is defined as the last subject's last visit.

In case the study is ended prematurely, the sponsor will notify the accredited METC and the competent authority within 15 days, including the reasons for the premature termination.

Within one year after the end of the study, the investigator/sponsor will submit a final study report with the results of the study, including any publications/abstracts of the study, to the accredited METC and the Competent Authority.

The investigator will submit any proposed publication or presentation along with the respective scientific journal or presentation forum at least 7 days prior to submission of the publication or presentation to all co-investigators.

9.3 Joint Investigator/Sponsor Responsibilities

9.3.1 Access to Information for Monitoring

In accordance with International Conference on Harmonisation Good Clinical Practice (ICH-GCP) guidelines, the study monitor must have direct access to the investigator's source documentation in order to verify the data recorded in the CRFs for consistency.

The monitor is responsible for routine review of the CRFs at regular intervals throughout the study, to verify adherence to the protocol, and the completeness, consistency and accuracy of the data being entered on them. The monitor should have access to any subject records needed to verify the entries on the CRFs. The investigator agrees to cooperate with the monitor to ensure that any problems detected in the course of these monitoring visits are resolved.

9.3.2 Study Discontinuation

The investigator reserves the right to terminate the study at any time. Should this be necessary, the investigator will arrange discontinuation procedures. In terminating the study, the investigator will assure that adequate consideration is given to the protection of the subjects' interests.

9.4 Facilities

The study will be performed at the hospitals listed under 4.1. Drug will be supplied by Takeda.

10. ADMINISTRATIVE ASPECTS, MONITORING AND PUBLICATION

10.1 Handling and storage of data and documents

The investigator must assure that subjects' anonymity will be strictly maintained and that their identities are protected from unauthorized parties (see 10.1.6 Confidentiality). Blood samples for pharmacokinetic analysis will be identified by study name, date and time of sampling and sample code. The medical record and other source documents are only accessible by the medical staff of the clinical research centre, the investigator and monitor as well as for audits by METC members or authorized government personnel. CRFs do not contain identifiable information and will be coded with subject ID numbers only. Information and study files that are necessary for the evaluation of the research are stored anonymously and the identification key will not be accessible by unauthorized parties.

10.2 Monitoring and Quality Assurance

The monitor will be a staff member at the Pharmacy at the Radboud university medical centre. The monitor will work independently and has no involvement in the set up of the study, the conduct of the study and interpretation of the results.

Prior to first screening an initiation visit will be performed with the site and after the trial a close out visit will be performed with the site. The monitor will work according to Monitoring Guidelines and Checklists that will be written especially for this study.

Monitoring consists of:

- Check essential documents at the site
- Check eligibility of subjects prior to study start
- Monitoring for completeness and correctness of the source documents
- Monitoring of the data in the workbook, and transfer of data from source documents to the CRF
- Write monitoring reports

The investigator should understand that source documents for this study should be made available to regulatory authority or health authority inspectors.

See also 'Access to information for monitoring'.

10.3 Amendments

A 'substantial amendment' is defined as an amendment to the terms of the METC application, or to the protocol or any other supporting documentation, that is likely to affect to a significant degree:

- the safety or physical or mental integrity of the subjects of the trial;
- the scientific value of the trial;
- the conduct or management of the trial; or
- the quality or safety of any intervention used in the trial.

All substantial amendments will be notified to the METC and to the competent authority.

Non-substantial amendments will not be notified to the accredited METC and the competent authority, but will be recorded and filed by the sponsor.

10.4 Annual progress report

The sponsor/investigator will submit a summary of the progress of the trial to the accredited METC once a year. Information will be provided on the date of inclusion of the first subject, numbers of subjects included and numbers of subjects that have completed the trial, serious adverse events/ serious adverse reactions, other problems, and amendments.

10.5 End of study report

The sponsor will notify the accredited METC and the competent authority of the end of the study within a period of 90 days. The end of the study is defined as the last subject's last visit.

In case the study is ended prematurely, the sponsor will notify the accredited METC and the competent authority within 15 days, including the reasons for the premature termination.

Within one year after the end of the study, the investigator/sponsor will submit a final study report with the results of the study, including any publications/abstracts of the study, to the accredited METC and the Competent Authority.

10.6 Public disclosure and publication policy

The study will be registered to a publicly accessible registry and results database (ClinicalTrials.gov). The investigator will submit any proposed publication or presentation along with the respective scientific journal or presentation forum at least 7 days prior to submission of the publication or presentation to all co-investigators. See 10.2.3 study reports and publication.

11. Reference List

All references are provided as in text references
