# Supplementary material for: Intravenous lanadelumab for the treatment of moderately ill COVID‐19 patients
Source: Br J Clin Pharmacol. 2026 Jan 9;92(6):1685–95. doi: 10.1002/bcp.70438 (PMC13206366; doi:10.1002/bcp.70438)
Supplement: Supplementary file 2 — Data S2. Supporting Information. [file BCP-92-1685-s001.docx]

**Supplementary Methods.**

**Randomization and trial interventions.** During this trial, multiple blood samples were taken from participants in both randomization groups. The analyses performed with these samples are not reported in this trial, but a detailed description of sampling is given here. Clinical laboratory values, comprising hemoglobin, leukocyte count and differentiation, platelet count, C-reactive protein, ferritin, D-dimer, albumin, alanine aminotransferase, and aspartate aminotransferase, were measured at baseline and daily afterwards. On days one and four of the trial, additional blood samples were taken for pharmacodynamic analyses. In the lanadelumab group only, blood samples for pharmacokinetic analyses were taken daily.

A protocol aimed at active reduction of supplemental oxygen was used for all patients. The target peripheral oxygen saturation (measured using pulse oximetry) for all patients was set at ≥93%. When, at any point during the trial, a peripheral oxygen saturation of ≥97% was measured, the supplemental oxygen volume for that patient was incrementally lowered in steps of one L/min. After an hour, peripheral oxygen saturation was measured again, and supplemental oxygen was reduced further if peripheral oxygen saturation remained ≥97%. When a peripheral oxygen saturation below 93% was measured, supplemental oxygen was increased as needed in order to reach the target saturation of ≥93%.

For safety considerations, a ramp-up scheme was used for lanadelumab administrations. The drug was administered over the course of one hour, increasing the rate of infusion after every 20 minutes (90 mg/hour; 210 mg/hour; 600 mg/hour). During lanadelumab administration and throughout the trial’s observational period, patients in both treatment groups were closely monitored for adverse effects. Nursing staff administering the study drug were instructed to have the patients report any and all side effects and symptoms occurring since the treatment was started and whenever the rate of infusion was increased. Furthermore, patients were visited daily by an attending physician, and laboratory values were monitored daily for the occurrence of adverse effects.

**Pharmacokinetic sampling and analyses.** Study participants in the intervention group were included for pharmacokinetic (PK) assessment. Blood samples were to be collected at predose (dose 1) and at the following times postdose: 12 (Day 1), 24 (Day 2), 48 (Day 3), 72 (predose dose 2), 96 (Day 5), 120 (Day 6), and 144 (Day 7) hours postdose, and then daily (i.e., 168 (Day 8), 192 (Day 9) hours postdose). Samples for determination of plasma lanadelumab were drawn in SCAT-169 tubes.

Patients who received at least the first lanadelumab dose and at least one evaluable PK concentration postdose were included in the PK descriptive analysis. All PK parameters of lanadelumab were summarized with descriptive statistics (number of observations, arithmetic mean, standard deviation, minimum, median, maximum, percent coefficient of variation, geometric mean, and/or percent coefficient of variation of the geometric mean). Only number of observations, minimum, median, and maximum were reported.

Lanadelumab was determined using a validated Enzyme Linked Immunosorbent Assay (ELISA) for the quantitation in human SCAT-169 tubes. The lower limit of quantification (LLOQ) of the assay found to be 3.13 ng/mL at the maximum repeatable dose (MRD) with an accuracy of -35% of recovery (% RE) and an imprecision of 16.8%. The upper limit of quantification (ULOQ) of the assay was found to be 400 ng/mL at the MRD with an accuracy of -5/3% RE and imprecision of 12.9%. Quality control samples were found to be stable for a minimum of 4 hours at room temperature. The assay showed acceptable recovery with values ranging from 81.6-101.5% of their nominal values. The assay demonstrated robustness to time at all conditions tested. Finally, the assay demonstrated dilutional linearity with % RE values ranging from -7.7% to 14.1% of the nominal values and coefficient of variation (CV) values ranging from 0.0 – 3.6%.
