# Supplementary material for: Intravenous lanadelumab for the treatment of moderately ill COVID‐19 patients
Source: Br J Clin Pharmacol. 2026 Jan 9;92(6):1685–95. doi: 10.1002/bcp.70438 (PMC13206366; doi:10.1002/bcp.70438)
Supplement: Supplementary file 3 — Data S3. Supporting Information. [file BCP-92-1685-s002.docx]

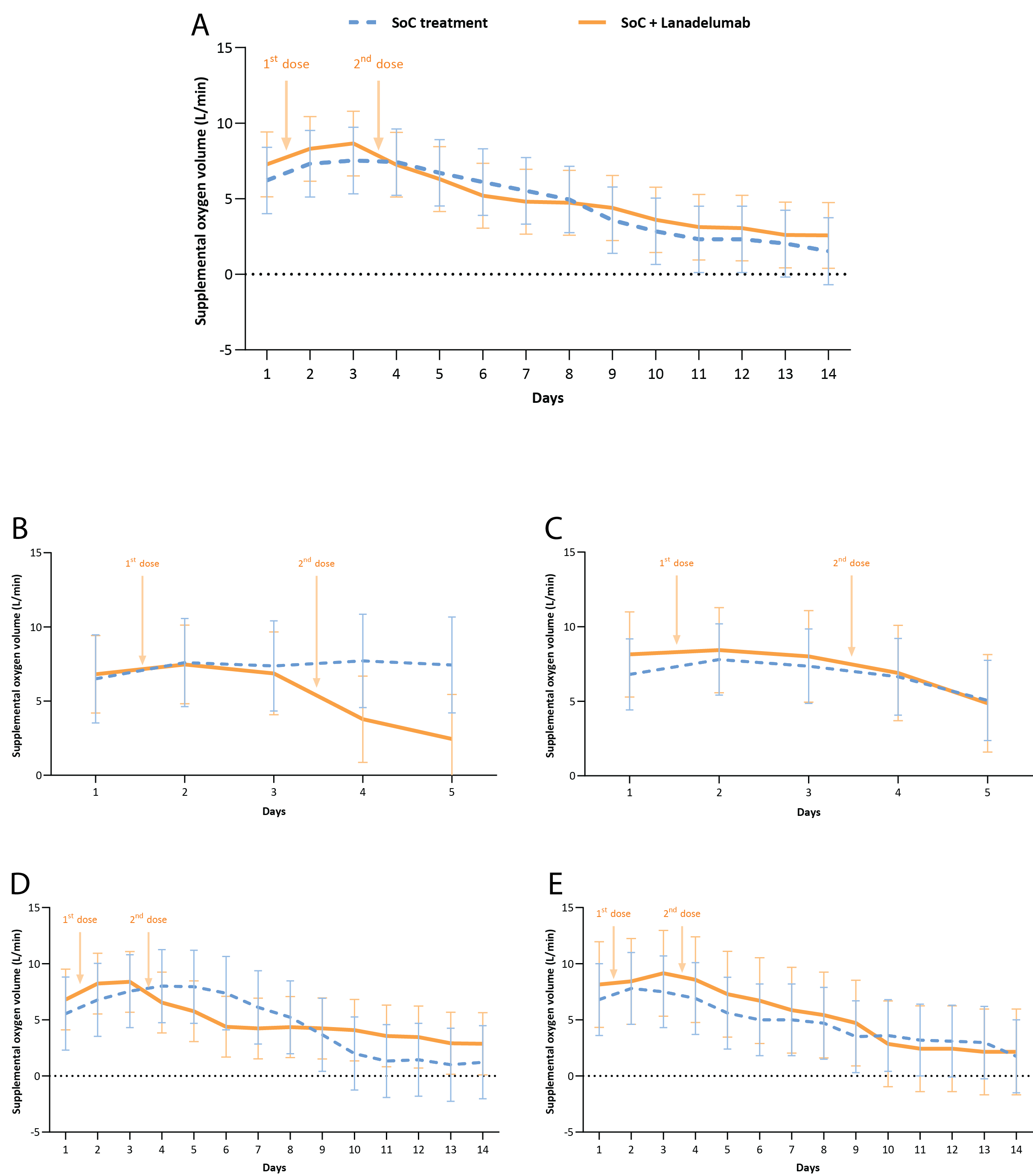


**Supplemental Figure 1.**

1. Predicted supplemental oxygen volumes over the full 14-day follow-up period of the trial. Missing data were handled using worst-case imputation, where patients receiving high-flow oxygen therapy or mechanical ventilation were assigned 15 L/min of oxygen, patients who had been discharged from the hospital without supplemental oxygen were assigned 0 L/min, and patients discharged with supplemental oxygen were assigned 1 L/min. Oxygen volumes were predicted with linear mixed model analysis, where treatment group, time, and an interaction term of time x treatment group were entered as fixed effects. Neither treatment group nor interaction between treatment group and time significantly predicted supplemental oxygen volumes (p = 0.76 and p = 0.86, respectively).
2. Predicted supplemental oxygen volumes over the first five days of the trial in patients who had been enrolled between 4-10 days after symptom onset (n = 23; 10 in the control group and 13 in the intervention group). While treatment group did not significantly affect supplemental oxygen volumes, interaction between treatment group and time did (p = 0.32 and p = 0.04, respectively).
3. Predicted supplemental oxygen volumes over the first five days of the trial in patients who had been enrolled between 11-20 days after symptom onset (n = 17; 10 in the control group and 7 in the intervention group). Neither treatment group nor interaction between treatment group and time significantly predicted supplemental oxygen volumes (p = 0.73 and p = 0.97, respectively).
4. Predicted supplemental oxygen volumes over the full 14-day follow-up period in patients who had been enrolled between 4-10 days after symptom onset (n = 23; 10 in the control group and 13 in the intervention group), using the worst-case imputation method described above to handle missing primary outcome data. Neither treatment group nor interaction between treatment group and time significantly predicted supplemental oxygen volumes (p = 0.85 and p = 0.15, respectively).
5. Predicted supplemental oxygen volumes over the full 14-day follow-up period in patients who had been enrolled between 11-20 days after symptom onset (n = 17; 10 in the control group and 7 in the intervention group), using the worst-case imputation method described above to handle missing primary outcome data. Neither treatment group nor interaction between treatment group and time significantly predicted supplemental oxygen volumes (p = 0.76 and p = 0.91, respectively).
