# Supplementary material for: Intravenous lanadelumab for the treatment of moderately ill COVID‐19 patients
Source: Br J Clin Pharmacol. 2026 Jan 9;92(6):1685–95. doi: 10.1002/bcp.70438 (PMC13206366; doi:10.1002/bcp.70438)
Supplement: Supplementary file 4 — Data S4. Supporting Information. [file BCP-92-1685-s003.docx]

| **Descriptive statistics of PK parameters of lanadelumab in participants included in the intervention group** | | | |
| --- | --- | --- | --- |
| **Descriptive Statistics** | **AUC (µg*day/mL)** | **C_max_1 (µg/mL)** | **C_max_2 (µg/mL)** |
| N | 16 | 16 | 16 |
| Mean | 1301 | 69.3 | 96.7 |
| SD | 129 | 55.5 | 57.2 |
| CV% | 9.9 | 80.2 | 59.1 |
| Min | 1088 | 19.8 | 34.8 |
| Median | 1281 | 50.8 | 78.9 |
| Max | 1525 | 264 | 288 |
| Geometric Mean | 1295 | 58.4 | 86.6 |
| Geometric CV% | 10.0 | 59.1 | 47.7 |

AUC = Area under the curve from time zero to infinity

C_max_ = Maximum observed plasma drug concentration following the first IV dose (C_max_1) and following the second IV dose (C_max_2);

CV% = Coefficient of variation
